# Gut microbiome in two high-altitude bird populations showed heterogeneity in sex and life stage

**DOI:** 10.1093/femsmc/xtae020

**Published:** 2024-07-04

**Authors:** Mingwan Sun, Naerhulan Halimubieke, Baozhu Fang, José O Valdebenito, Xieyang Xu, Samuel K Sheppard, Tamás Székely, Tongzuo Zhang, Shunfu He, Rong Lu, Stephen Ward, Araxi O Urrutia, Yang Liu

**Affiliations:** State Key Laboratory of Biocontrol, School of Life Sciences/School of Ecology, Sun Yat-sen University, Guangzhou 510275, China; Milner Centre for Evolution, Department of Life Science, University of Bath, Bath BA27AY, United Kingdom; Milner Centre for Evolution, Department of Life Science, University of Bath, Bath BA27AY, United Kingdom; Department of Anthropology, University College London, London WC1H 0BW, United Kingdom; State Key Laboratory of Biocontrol, School of Life Sciences/School of Ecology, Sun Yat-sen University, Guangzhou 510275, China; State Key Laboratory of Desert and Oasis Ecology, Xinjiang Institute of Ecology and Geography, Chinese Academy of Sciences, Urumqi 830011, China; Milner Centre for Evolution, Department of Life Science, University of Bath, Bath BA27AY, United Kingdom; Bird Ecology Lab, Instituto de Ciencias Marinas y Limnológicas, Universidad Austral de Chile, Independencia 631, Valdivia 5110566, Chile; Instituto Milenio Biodiversidad de Ecosistemas Antárticos y Subantárticos (BASE), Santiago 8331150, Chile; State Key Laboratory of Biocontrol, School of Life Sciences/School of Ecology, Sun Yat-sen University, Guangzhou 510275, China; Ineos Oxford Institute, University of Oxford, Oxford OX1 3RE, United Kingdom; Milner Centre for Evolution, Department of Life Science, University of Bath, Bath BA27AY, United Kingdom; Department of Evolutionary Zoology and Human Biology, University of Debrecen, Debrecen 4032, Hungary; Key Laboratory of Adaptation and Evolution of Plateau Biota, Northwest Institute of Plateau Biology, Chinese Academy of Sciences, Xining 810008, China; Qinghai Provincial Key Laboratory of Animal Ecological Genomics, Xining 810008, China; Xining National Terrestrial Wildlife Epidemic Monitoring Station, Xining 810008, China; Xining National Terrestrial Wildlife Epidemic Monitoring Station, Xining 810008, China; Department of Life Science, University of Bath, Bath BA27AY, United Kingdom; State Key Laboratory of Biocontrol, School of Life Sciences/School of Ecology, Sun Yat-sen University, Guangzhou 510275, China; State Key Laboratory of Biocontrol, School of Life Sciences/School of Ecology, Sun Yat-sen University, Guangzhou 510275, China

**Keywords:** gut microbiota, 16sRNA gene, shorebirds, environment, social structure, disease biology, mating system, development

## Abstract

Gut microbiotas have important impacts on host health, reproductive success, and survival. While extensive research in mammals has identified the exogenous (e.g. environment) and endogenous (e.g. phylogeny, sex, and age) factors that shape the gut microbiota composition and functionality, yet avian systems remain comparatively less understood. Shorebirds, characterized by a well-resolved phylogeny and diverse life-history traits, present an ideal model for dissecting the factors modulating gut microbiota dynamics. Here, we provide an insight into the composition of gut microbiota in two high-altitude (ca. 3200 m above sea level) breeding populations of Kentish plover (*Charadrius alexandrinus*) and Tibetan sand plover (*Charadrius altrifrons*) in the Qinghai–Tibetan Plateau, China. By analysing faecal bacterial communities using 16S rRNA sequencing technology, we find a convergence in gut microbial communities between the two species, dominated by *Firmicutes, Proteobacteria*, and *Bacteroidetes*. This suggests that the shared breeding environment potentially acts as a significant determinant shaping their gut microbiota. We also show sex- and age-specific patterns of gut microbiota: female adults maintain a higher diversity than males, and juveniles are enriched in *Rhizobiaceae* and *Exiguobacterium* due to their vegetative food resource. Our study not only provides a comprehensive descriptive information for future investigations on the diversity, functionality, and determinants of avian microbiomes, but also underscores the importance of microbial communities in broader ecological contexts.

## Teaser

Using 16s rRNA characterize the gut microbiome community in two plover species in high-altitude population to show difference on sex and ages.

## Introduction

Microbiome research is an emerging field in ecology and evolutionary biology. The gut microbiota, is a diverse community of bacteria, archaea, or eukaryotes that reside within the gastrointestinal tract, influencing the physiology, behaviour, and fitness of the host (Ley et al. 2008, Kohl 2012, Waite and Taylor 2014, Escallón et al. 2017). Being capable of powered flight and wide distribution, the diversity on morphology and physiology make avian species occupy a plenty of niches in ecosystem (Pigot et al. 2020) and also associated with more diverse microbiome environment (Waite and Taylor 2014). Changes on environment like urbanization will influence the diversity and composition of wild-bird microbiomes (Waite and Taylor 2014, Teyssier et al. 2020), also increase the colonization of pathogens (Murray et al. 2020), which may have the risk of human infection (Smith et al. 2020) and carry antimicrobial-resistance genes (Marcelino et al. 2019, Cao et al. 2020). Therefore, the investigate of avian gut microbiome contribute to both conservation and public health.

Several studies have investigated the factors that shape gut microbiota, with host phylogeny, environment, sex, and development being identified as important (Foster et al. 2017, Grond et al. 2018, McDonald et al. 2018, Watson et al. 2019). Gut microbial communities exhibit greater similarity among conspecific individuals than among individuals of different species (Ley et al. 2008, Goodrich et al. 2014, Waite and Taylor 2014, Hird et al. 2015). The environment, however, appears to have a significant effect. For instance, individuals from sympatric species (e.g. migratory passerines, chimpanzees, and gorillas; Moeller et al. 2013) show convergent gut microbial communities reflecting their similar ecology, environment, and diet. Conversely, individuals from populations at different locations can show divergent gut microbiota. For example, onshore polar bears exhibit significantly higher gut bacterial diversity compared to their offshore counterparts (Watson et al. 2019). Furthermore, there is evidence that behaviour like migration will lead to elevated abundance of certain bacterial genera in sandpipers (Risely et al. 2018). Sex can also be a factor since the gut microbiota of males and females differ in several species including humans (Elderman et al. 2018, Kim et al. 2020).

Sex differences can result from behavioural differences between males and females and primarily stem from intrinsic physiologic. In the former case, gut microbiota patterns in males and females should vary based on behavioural differences in species: some studies has shown that social contact can mediate the acquisition and flow of microbiomes between individuals (Degnan et al. 2012, Song et al. 2013, Nuriel-Ohayon et al. 2016). In the latter case, it could be expected that patterns of difference between males and females should be consistent across species. Most studies have suggested that the sex differences in gut microbiota stem from sex-dependent physiological conditions (e.g. sex hormone and body mass index) and diet (Dominianni et al. 2015, Neuman et al. 2015, Org et al. 2016, Escallón et al. 2017). Previous research has demonstrated that sex hormones may correlate with gut microbiota community diversity and the abundance of specific genera in both domesticated and wild animals (Flores et al. 2012, Org et al. 2016, Escallón et al. 2017, Wu et al. 2022). In domesticated mice and cows, hormone treatment shift the structure and composition of intestinal bacterial communities, suggesting the influence of hormones on the microbiome through metabolic processes and the immune system (Org et al. 2016, Wu et al. 2022) In wild male rufous-collared sparrows (*Zonotrichia capensis*), individuals with higher testosterone levels were more likely to be exposed to the risk of infection and more complex bacterial environment, therefore leads a higher diversity of microbiota community in breeding season (Escallón et al. 2017).

Several studies have reported changes in gut microbiome as the host develops (Cox et al. 2012, Faith et al. 2013, Grond et al. 2017, Videvall et al. 2018). For instance, gut microbial communities during early life in the young display substantial variability in diversity and abundance and are markedly different from gut microbiotas of conspecific adults (González-Braojos et al. 2012, van Dongen et al. 2013, Waite and Taylor 2014). Nevertheless, the mechanism behind age-related gut microbial difference is poorly studied, such differences can be caused by parenting behaviour (e.g. biparental care versus uniparental care). In several species, there is evidence of vertical transmission from mother to offspring playing an important role in the establishment of gut microbiotas during early life offspring (Nuriel-Ohayon et al. 2016). Another line of the alteration in microbiota community in the early stage would be the food resource (Michl et al. 2017, Prince et al. 2019, Zhu et al. 2021, Xu et al. 2023). In several studies, with the growth of juveniles, changes on diet can significantly influence the stability of the microbiota community structure (Michl et al. 2017, Prince et al. 2019, Zhu et al. 2021, Xu et al. 2023). In the red-crowded crane (*Grus japonensis*) and the crested ibis (*Nipponia nippon*), the introduction of high-protein food resource will increase the certain of several bacterial, and as the development, the diversity of the community will declines, suggesting a stable gut microbiome environment (Michl et al. 2017, Prince et al. 2019, Zhu et al. 2021, Xu et al. 2023). Besides the external factors, the development of immune system as a physiological factor can also influence the community of gut microbiome during the growth and aging (Badal et al. 2020, Zhang et al. 2022). In addition, during the growth period, changes in gut morphology can also modify the gut microbiome community, especially in amphibians undergoing metamorphosis (Zhang et al. 2020).

However, previous findings were mostly centring around mammalian species, studies of nonmammalian species are more limited, especially in shorebirds (sandpipers, plovers, and allies). Shorebirds breed on all continents and are highly diverse in life history, ecology, and behaviours (Székely 2019), thus representing a good system to disentangle the factors that control gut microbiota. A recent study on Arctic shorebirds shows that the local environment has greater impact than phylogeny on gut microbiota composition in wild, migratory birds under natural conditions (Grond et al. 2019). To promote our understanding of gut microbiome in shorebirds, we focus on two *Charadrius* plover species, *Charadrius alexandrinus* (Kentish plover, KP) and the recently *Charadrius altrifrons* (Tibetan sand plover, TSP) (Wei et al. 2022), to understand how host phylogeny, sex, and development relate to gut microbiota composition. These two species are ideal as they share similar life history traits and breeding habitat. Both KP and LSP are migratory, socially monogamous, biparental care, and they both breed at high-altitude Qinghai Lake from May to July (see the section ‘Methods’ for more information about study site) (Su et al. 2020). Therefore, the investigation on these two plover populations will assist understanding the phylogenetic effect on gut microbiome as well as how the microbiome in host contribute to adapting oxygen deficiency and extreme low temperature of local environment. The other, these two species still keep discriminant preference on habits like nesting site and foraging site: KP prefer to activate and nest along the coast of the Qinghai lake while TSP prefer the grassland (Halimubieke et al. 2024). This preference also provides an insight on the association between behaviour and difference on microbiome composition in these two species.

Here, we analyse microbial biodiversity in faecal samples collected from two wild populations of KP and TSP to assess the roles of species, sexes, and age groups in shaping the gut microbiota. We focus on exploring three main hypotheses to understand gut microbiota diversity and composition at different scales. First, we hypothesize gut microbiota composition to be convergent between both species due to the shared life history and breeding environment (Rothschild et al. 2018, Grond et al. 2019). We expect the main structure of gut microbial communities was similar in these two species but the microbiome in a more general taxonomy level (i.e. phylum) would be divergent among the individuals. Second, we expect sex-specific microbiomes as we speculate that physiological differences between the sexes influence gut microbiota composition and diversity (Bolnick et al. 2014, Kim et al. 2020, Hall et al. 2021). And microbiome highly abundant or insufficient in specific sex would be identified. Third, we predict differences in gut microbiota between adults and juveniles due to the intrinsic differences in the physiology of the two life stages (Waite and Taylor 2014, Zhu et al. 2021). We expect the microbiome diversity would be low because they neither had developed mature digest system nor had been equipped with foraging experience to diversify their food resource (Grond et al. 2017, Kozik et al. 2022). And the specific diet-related microbiome would be identified in juveniles as they lead a different diet and gut morphology.

## Materials and methods

### Study site and sample collection

Fieldwork was carried out at the Qinghai Lake (37°00′N 100°08′E), an alkaline lake on the Tibetan Plateau, in May and June of 2019. Qinghai Lake (36°50′30.78ʺN, 100°44′37.92ʺE) is the largest lake in China, located on the Qinghai–Tibetan Plateau with an elevation of 3200 m above sea level, where the temperature the average temperature in May and June is around 10.6°C and the oxygen concentration is low (Shi et al. 2021, Cedar Lake Ventures 2023). KPs and TSPs breed along the lake shore (Song et al. 2020). Breeding pairs were captured on their nest while incubating eggs, using funnel traps (Székely et al. 2008). The sex was determined by morphological characters in adults, and molecular sexing in chicks (see Que et al. 2019). Gut microbiota samples were collected from 24 captured KPs (7 adult males, 10 adult females, 5 male chicks, and 2 female chicks) and 9 TSPs (2 adult males, 5 adult females, 1 male chick, and 1 female chick). Faecal samples are generally representative of the bacterial community in the large intestine (Yan et al. 2019). We adopted faecal sampling protocol in Knutie and Gotanda (2018). The captured bird was put into a paper bag with sterile wax paper on the bottom. A metal grate was set over the wax paper to prevent the bird from directly encountering the faeces after defecation. After defecation, faecal samples were collected using sterile polyester swabs, placed in sterile cryotubes without medium, and kept in liquid nitrogen. The grate was sterilized by soaking them in a 10% bleach solution for at least 10 min before each collection to reduce potential cross-contamination between different individuals. To minimize stress, birds were held for no longer than 15 min before being released. Samples were transported in liquid nitrogen to a laboratory freezer at −80°C. As studies suggest, differences in bacterial composition resulting from storage conditions do not eclipse differences between samples, even when left at ambient temperatures for 2 weeks (Lauber et al. 2010, Dominianni et al. 2014, Song et al. 2016), we assume that changing of the storage environment had minimal effect on microbial composition of the samples collected.

### DNA isolation, amplification, and sequencing

To obtain enough genomic DNA for sequencing library preparation, the faecal sample on the sterile tips was used to extract genomic DNA with the FastDNA Spin Kit for Faeces (MP Biomedicals Co., Ltd., USA) following the manufacturer’s instructions. Then, the concentration and purity of the extracted DNA samples were measured using a Nanodrop 2000 spectrometer (Thermo Fisher Scientific, Wilmington, DE, USA). 16S rRNA gene libraries were amplified from DNA extracts using PCR (Polymerase Chain Reaction) primers 515F (5-GTGYCAGCMGCCGCGGTAA-3) and 806R (5-GGACTACNVGGGTWTCTAAT-3), targeting the variable region 4 of the 16S rRNA gene (Caporaso et al. 2012). The 250-bp paired-end amplicons are then combined and sequenced on an Illumina Miseq platform in two separate runs. The quality control of the sequencing data was performed using the VSEARCH (version 1.9.6) platform (Rognes et al. 2016). Paired-end reads were merged and reads paired less than 20 bp or containing unknown base calls were discarded. Then the PCR amplification primers were trimmed, and the sequences with length greater than 200 bp were kept for further quality control, and those displaying greater than 0.5% Expected Error were discarded. Using the VSEARCH (version 1.9.6) platform (Rognes et al. 2016), chimera sequence were removed according to the chimera annotation in the Ribosomal Database Program (RDP) (Cole et al. 2014). Operational taxonomic units (OTUs) were created by clustering sequences with 97% sequence identity, discarding chimeric sequences after being aligned to the SILVA reference (Pruesse et al. 2007). Taxonomic assignments of representative sequences from each OTU were performed using the RDP classifier (version 2.2) (Cole et al. 2014). Sequences with ≥97% similarity were assigned to the same OTU. Our rarefaction analysis shows the result of our sequencing data sufficiently represents the community (Supplementary Figure 1).

### Statistical analyses

All the faecal samples were classified into three groups: (a) species (KP versus TSP); (b) sex (female versus male); and (c) age (adult versus juvenile). To test our first hypothesis regarding convergence in species, we applied a statistical test to all samples categorized as 24 KP and 9 TSP. For the second hypothesis, we compared all samples in different sexes within the same age group: 9 males, 15 females in adults and 6 males, and 3 females in juveniles. Lastly, to discern differences in separate life stages despite limited samples, we compared 24 adults and 9 juveniles across all samples.

First, the shared core microbiome was identified by the OTUs presenting in 50% of all samples according to the previous study (Unterseher et al. 2011). Then, we analysed community diversity within each group by calculating α-diversity indices (Chao1, Shannon, and Simpson) using Qiime (version 1.9.1) under the rarefaction of 39 766 reads (Caporaso et al. 2010); then the ANOVA and Mann–Whitney U test was used to compare the differences within each comparison group, and the result of each combination in the comparison group was in Supplementary File 3.

We analysed the microbial composition dissimilarity within each comparison group (β-diversity) across both species. We used a principal coordinates analysis (PCoA) based on the Bray–Curtis distance matrices using all 4046 OTUs to discriminate the differences in microbial composition within each comparison group. To examine differences in β-diversity within each comparison group, 29 PcoA components are fitted with analysis of variance using distance matrices (Adonis, vegan package in R) (Dixon 2003), results showed in Supplementary File 3. In visualization, Linear discriminant analysis Effect Size (LEfSe) analysis is applied to discriminate the differential OTUs in each group category (trans_diff, microeco package in R) using the unweighted unifrac distance (Liu et al. 2021). OTU with LDA above 2 were selected for the PCoA. Network Analysis was applied to genus relative abundance data by weighted correlation network analysis (WGCNA). And differential expressed genus was identified by limma (Ritchie et al. 2015).

## Results

### OTU clusters and species abundance

**Figure 1.**
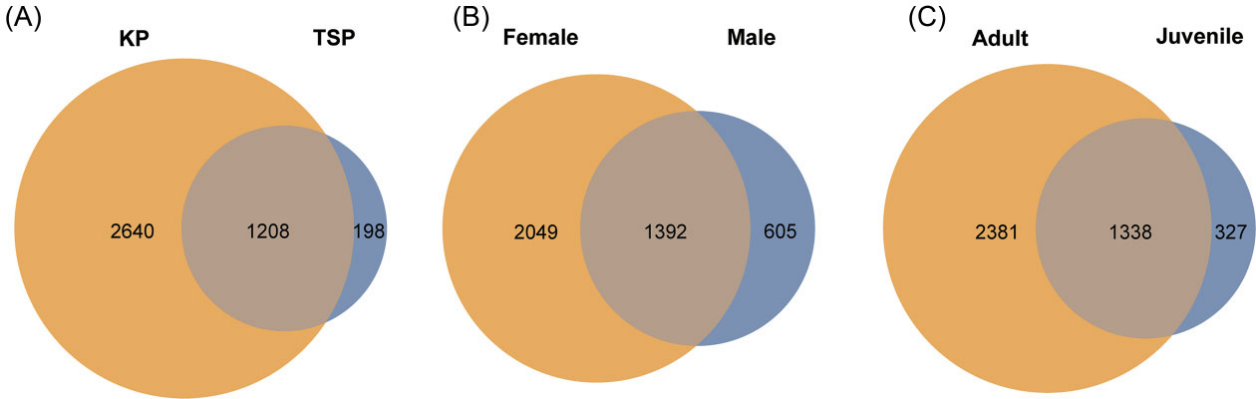
Venn diagrams demonstrating number of shared and unique OTUs in (A) KPs (left) and TSPs (right), (B) in female (left) and male (right) KP, and (C) in adult (left) and juvenile (blue) KPs.

A total of 5254 OTUs were identified from faecal samples of 24 KPs (17 adults and 7 juveniles) and nine TSPs (seven adults and two juveniles). Specifically, at the species level, the faecal microbiota of KPs comprised 3848 OTUs, contrasting with the 1406 OTUs observed in TSPs. Among these, 1208 OTUs were shared between the two populations (Fig. 1A). Of the total number of OTUs found, 2640 OTUs were unique in KPs, while a considerably smaller number of OTUs (*n* = 198) were specific to TSPs. Between the sexes, female plover faecal microbiota consisted of 3441 OTUs, and male plovers exhibited 1997 OTUs, as 1392 OTUs were shared between sexes (Fig. 1B). To be more specific, 2049 OTUs were unique to females, and 605 to males. Within the age group, the number of OTUs in adult and juvenile plover faecal microbiotas were 3719 and 1665, respectively. A total of 1338 OTUs were shared between adults and juveniles (Fig. 1C) and 2381 OTUs were unique to adults compared to 327 for juveniles. From the result, we expected that the variation of OTUs mainly comes from the bias on sample size: the group with more samples may contain more OTUs.

**Figure 2.**
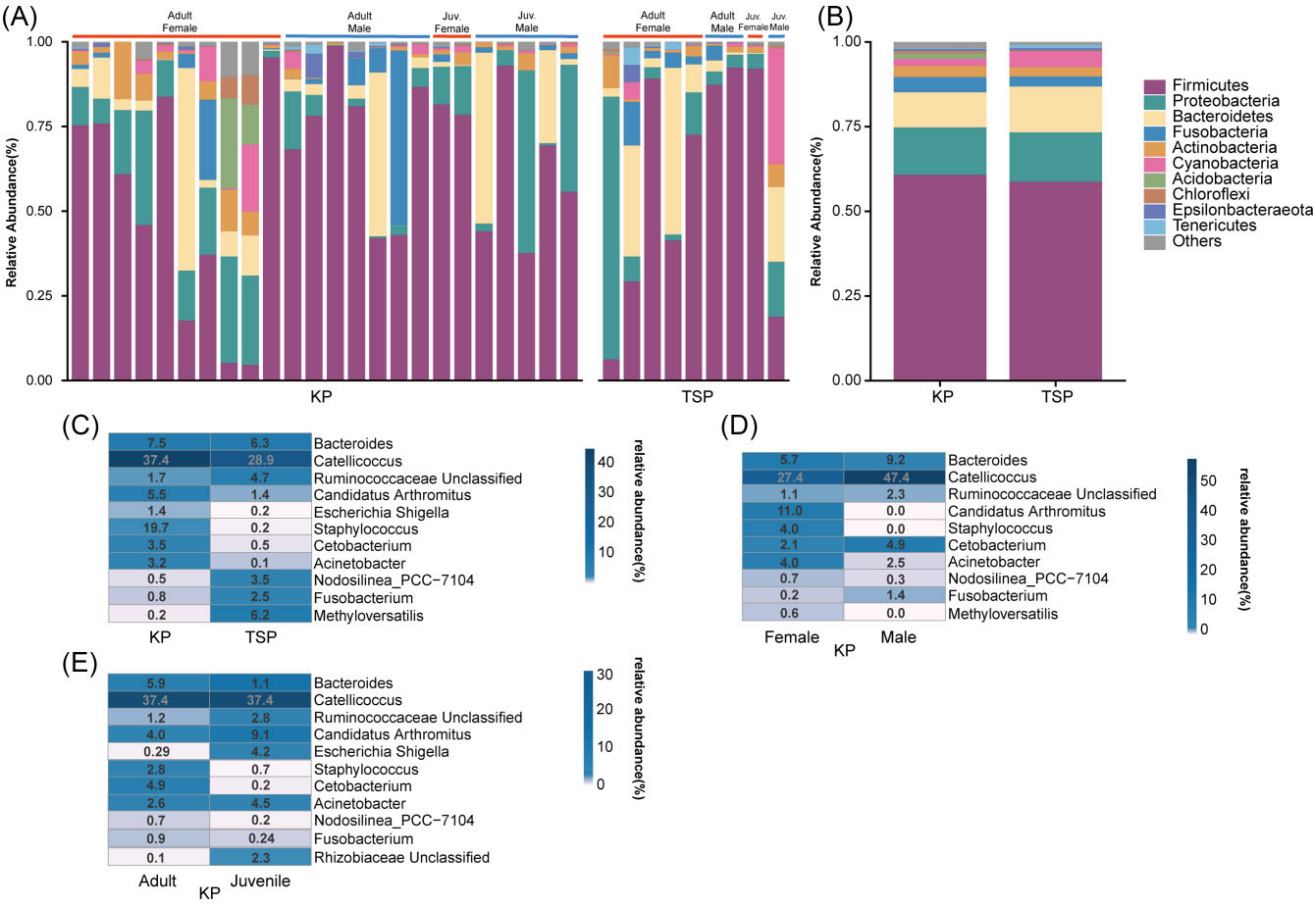
(A) Stacked bar chart of the relative abundance of top 10 bacterial phyla in the faecal microbiota of 24 KPs and 9 TSPs. Information of sample is shown above the each bar (Juv. indicates Juveniles). (B) Stacked bar chart of the average relative abundance of bacterial phyla in faecal microbiota of KPs and TSPs. Phyla in the legend are listed in order of decreasing abundance. (C) Heatmap of relative abundance of genus and species in faecal microbiota of KPs and TSP, female and male in KPs (D), and adult and juvenile in KPs (E).

At the species level, all the OTUs encompass 36 bacterial phyla, with the prevalence and abundance of specific phyla differing among individuals (Fig. 2A). The dominant phyla in both species showed consistency, with the most abundant phyla being Firmicutes (60% in KPs and 59% in TSPs), Proteobacteria (14% in KPs and 15% in TSPs), and Bacteroidetes (10% in KPs and 14% in TSPs) (Fig. 2B).

We observed discernible patterns provides insights into the distribution of microbial taxa between different species as shown in the heatmap (Fig. 2C). Notably, Bacteroides and Catellicoccus, co-occur in both KPs and TSPs. KPs showed a higher prevalence of *Candidatus* ‘Arthomitus’, *Escherichia*–*Shigella, Staphylococcus, Cetobacterium*, and Acinetobacter, while TSPs exhibited relative richness in an unclassified genus in *Ruminococcaceae, Fusobacterium, Methyloversatillis*, and a species in *Nodosilinea* (Fig. 2C). Upon closer investigation on the KP microbiome, we discerned sexual differences. Specifically, a female bias was observed in the abundance of *Candidatus* ‘Arthomitus’, Staphylococcus, and Acinetobacter; whereas Catellicoccus showed a relative male bias. In contrast, genera such as Bacteroides, the unclassified genus in *Ruminococcaceae*, and *Cetobacterium* exhibited no significant variance between sexes (Fig. 2D). Analysis of distinct age cohorts revealed a notable disparity, with adults displaying the enrichment of Staphylococcus and *Cetobacterium* compared to juveniles. Meanwhile, the increased prevalence of *Escherichia*–*Shigella* in KPs was predominantly observed in juvenile samples (Fig. 2E). Moreover, the discovery of an unconventional genus in *Rhizobiaceae*, known for its symbiotic affiliation with plant roots (Kaur and Vishnu 2022), as exclusive to juveniles suggested nuanced dietary distinctions among juveniles.

### Core microbiota

Here, the shared core microbiota of KPs and TSPs is defined by the cooccurrence in at least 50% of the samples. 92 OTUs were identified as shared core microbiota, among which 44 OTUs (47.8%) were assigned as the Phylum Firmicutes, separately to the Order *Clostridiales* (26%) and Order *Lactobacillales* (14.1%). The second abundant Phylum was *Proteobacteria* making up 29.3% of the shared core microbiota in 27 OTUs, mainly belonging to the Order *Rhodobacterales* (8.69%). Fewer OTUs were from Phylum *Bacteroidetes* (8.69%), *Actinobacteria* (5.43%), and *Cyanobacteria* (5.43%). The shared core OTUs displayed differentiation between the sexes (*R*^2^ = 0.0958, *P* = .044, envfit), not between the age groups (*R*^2^ = 0.0806, *P* = .067, envfit) and species (*R*^2^ = 0.046, *P* = .231, envfit).

### Differences in microbial α-diversity within comparison groups

**Figure 3.**
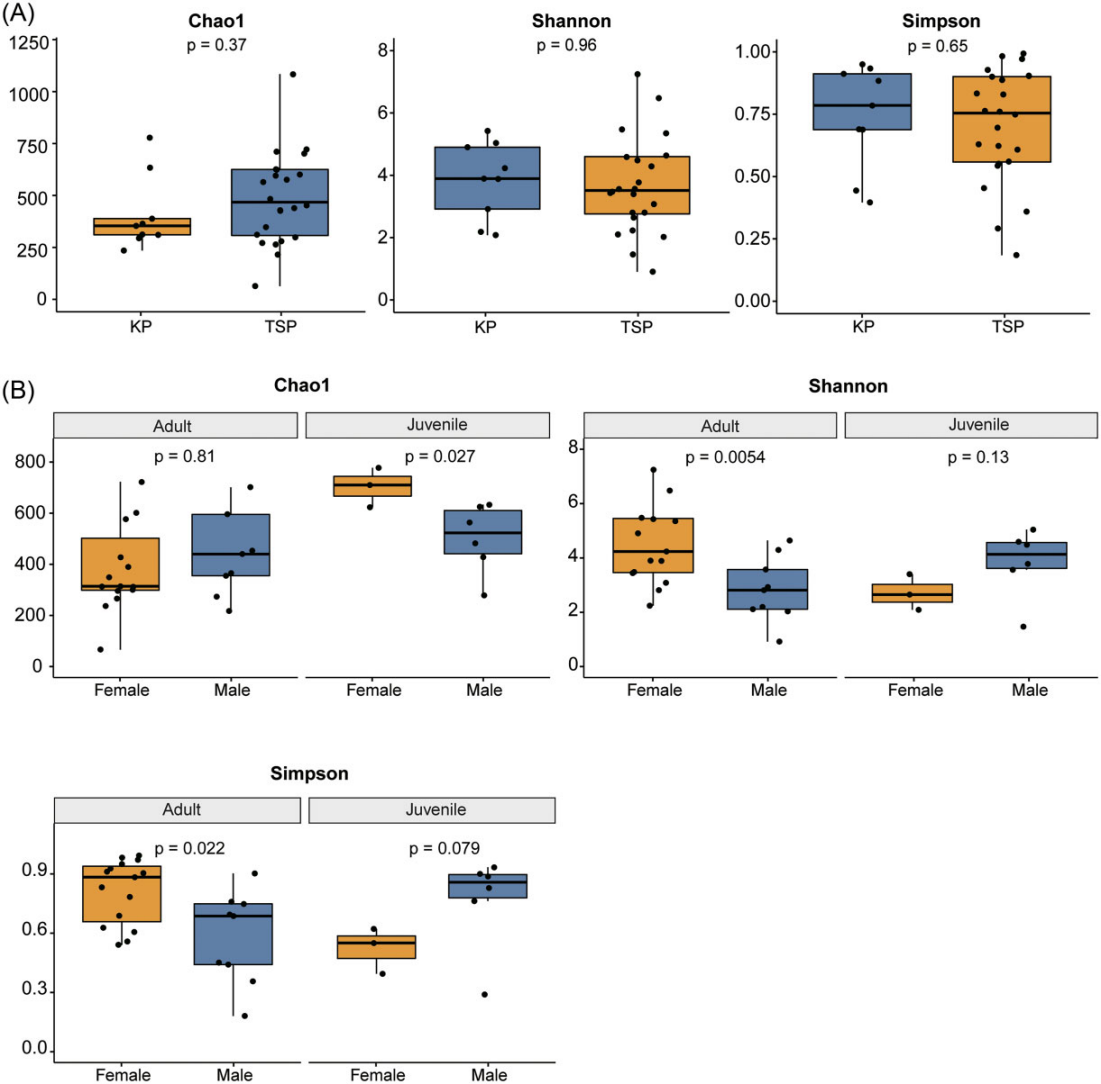
Chao1 diversity index, the Shannon diversity index, and the Simpson index of faecal samples of hosts of different species (A), and sex in same age group (B). Medians, upper, and lower quartiles are shown.

As estimates of microbial *α*-diversity, describing the diversity of the microbiome in each sample, we used the Chao1 diversity index, the Shannon diversity index, and the Simpson index (Supplementary Table 3). It was shown that α-diversity did not differ within each single comparison group (KP versus LSP, Female versus Male, and Adults versus Juvenile), (Fig. 3A, Supplementary Figure 2). The ANOVA test revealed a significant interaction between age and sex categories; however, no significance was observed when considering only species, age, and sex (Supplementary Table 4, Supplementary File 2). Among three α-diversity indexes, the difference between groups presented an opposite trend in the index considering both evenness and richness (Shannon and Simpson indices) with the index only considering the richness (Chao1 diversity index) (Fig. 3, Supplementary Figure 2). Nevertheless, a significant difference was observed between sexes in the same age group: adult females exhibited higher evenness than that in adult males, while the result reversed in the juvenile groups (Fig. 3B). When comparing the richness index, there was no significance shown in adult groups but in juvenile groups (Fig. 2B). Attractively, we found the sex diversity difference tendency has opposite direction: in adult group, female group has lower richness and higher evenness; and in juvenile group, female group has higher richness and lower evenness (Fig. 2B).

### Differences in microbial *β*-diversity and community-wide diversity within comparison groups

**Figure 4.**
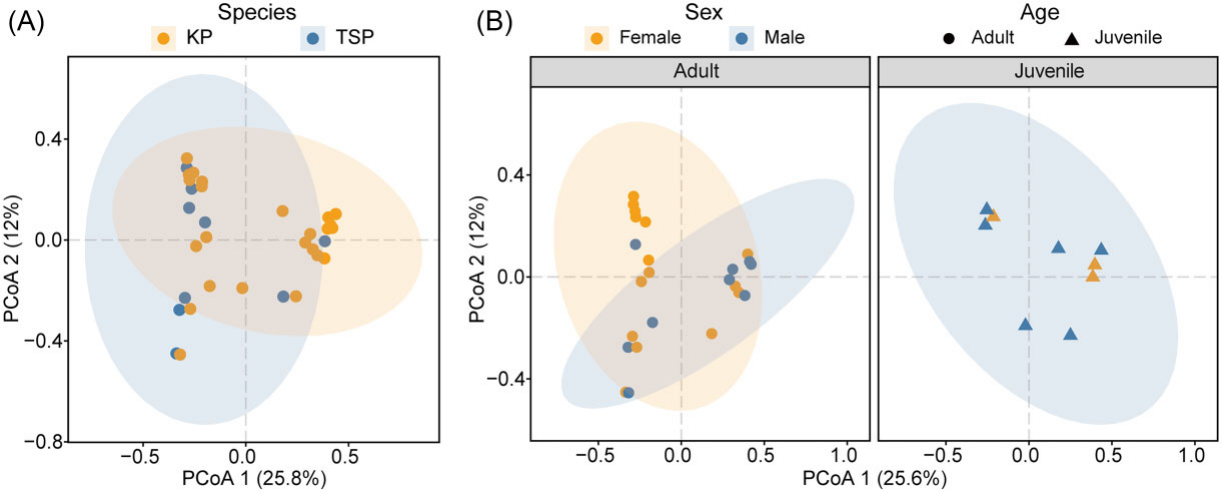
PCoA based on Bray–Curtis distance matrices showing the differences in microbial composition between (A) species and (B) sex difference in same age group.

Microbial composition dissimilarity between samples (*β*-diversity) was visualized using PCoA and tested by PERMANOVA. Our results showed that *β*-diversity showed a significant difference in age group and significant interaction between age and sex group when using the Bray–Curtis distance of 4046 OTUs (Supplementary Table 5). Like *α*-diversity, no difference was observed in species and sex group. For visualization, OTUs were selected by LEfSe analysis: 421 OTUs were used to show differences in species (Fig. 4A); 643 OTUs were used to show sexual differences in the same age group (Fig. 4B).

### Network analysis identified microbiome module enriched in juveniles

**Figure 5.**
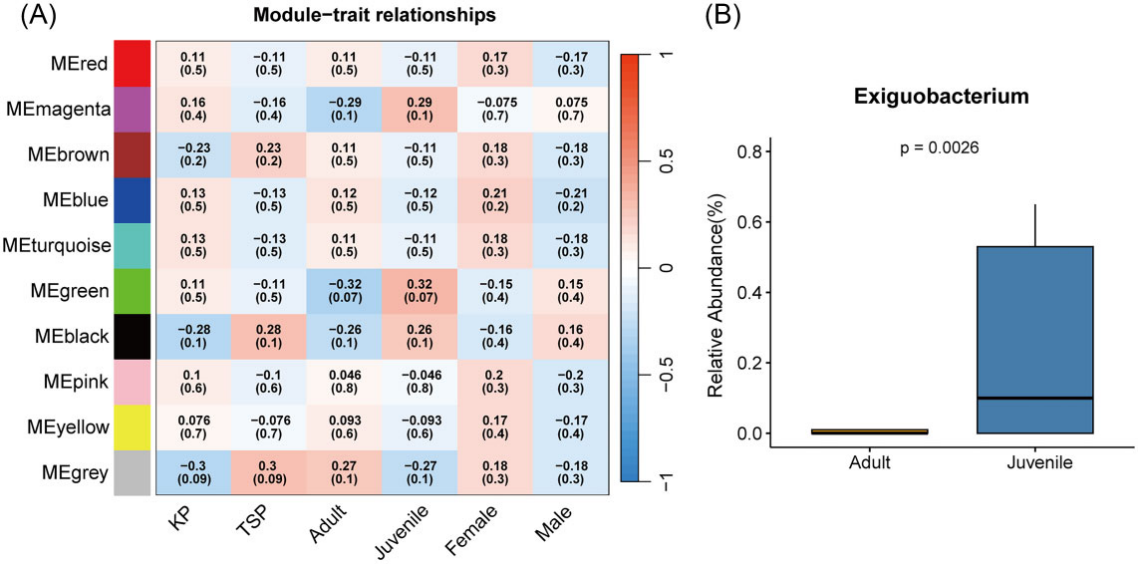
(A) WGCNA identified coexist microbiome module, correlation was calculated with each category, correlation coefficient is showed with the up number and the colour in each block, *P*-value is in the brackets. (B) Relative abundance of *Exiguobacterium*, differential abundance was tested using limma.

Through WGCNA, using filtered 168 highly expressed genus relative abundance data, 10 microbiome modules were identified (Fig. 5A). The microbiome module green was positively correlated with juvenile samples (cor = 0.32, *P* = .07) in which 12 genera were clustered (Fig. 5A, Supplementary Table 6). Among the 12 genera, we discovered the genus *Exiguobacterium* was significantly enriched (*P* = 0.0026, *q* = 0.032, limma) in juveniles (Supplementary Table 6, Fig. 5B). Some species in *Exiguobacterium* have already been proven to be plant growth-promoting bacteria and help accumulate selenium (Marfetán et al. 2023). The result showing the evidence that adult and juvenile may have different diet: juvenile prefer to more proportion of vegetations in their diet. Other previous study also suggested that juvenile individuals were insufficient of foraging experience to intake food with low trophic level (Durell 2003, Hall et al. 2021).

## Discussion

In this study, given the limited understanding of *Charadrius* plover gut microbiome, our initial investigation focused on elucidating the gut microbiome composition in two sympatric *Charadrius* plover populations breeding in a high-altitude environment. We found that at the phylum level, the most abundant phyla in KP and TSP faeces are *Firmicutes, Proteobacteria*, and *Bacteroidetes*, accounting for nearly 90% of the total gut microbiome. This result was consistent with previous studies of the gut microbiome in other avian species (Roggenbuck et al. 2014, Hird et al. 2015, Grond et al. 2018). *Firmicutes*, recognized for their association with mass gain and immune function in mammals and birds, play a pivotal role in augmenting nutrient uptake and metabolic efficiency (Flint et al. 2012, Li et al. 2015, John and Mullin 2016). *Bacteroidetes* is postulated to have a specific role in the breakdown of cellulose and other plant materials (Thomas et al. 2011, Kohl et al. 2014). However, the function of *Proteobacteria* in birds remains undetermined. It is also noteworthy that a considerable number of *Fusobacteria* were seen in KP (relative abundance 4.4%) and TSP (relative abundance 2.9%) faecal samples. *Fusobacteria* are often studied in the context of pathogenicity, and in carnivorous birds, along with an apparent beneficial role in the resistance against pathogens (Roggenbuck et al. 2014, Mendoza et al. 2018). The results highlight a high degree of consistency in the phylum composition and diversity between these two plover species. One plausible explanation would be, as these two species will choose the reproduction sites near the Qinghai Lake. The shared environment likely contributes to a uniform external microbiome, consequently influencing a similar gut microbiome community in these avian populations (Lewis et al. 2016, Grond et al. 2019).

At the genus level, our analysis revealed enrichment of certain genera across all categories or preferentially in specific categories, potentially influenced by distinct habits and diets. The genus *Bacteroides*, known to contain species acting as potential pathogens in both humans and birds (Thomas et al. 2011), displayed a relatively stable proportion in the gut microbiome composition of each group. This leads us to speculate that this genus may be associated with local environmental adaptation. *Catellicoccus* counts as the most abundant genus in each group (27.4%–47.4%) would be the dominant microbiome in plover gut. Consistent result have been reported in other waterbirds (Santos et al. 2012, Grond et al. 2014, Laviad-Shitrit et al. 2019). Several studies have employed species within this genus to assess faecal contamination in gulls (Ryu et al. 2012, Sinigalliano et al. 2013). Besides, we also identified some genus enriched in specific group decipher their difference on diet and habit. *Candidatus* ‘Arthomitus’ and *Cetobacterium* come from arthropods (Thompson et al. 2012) and fish (Li et al. 2015, Wang et al. 2021), respectively, which illustrate distinct diets between KP and TSP. Furthermore, *Methyloversatillis*, is primarily sourced from freshwater environments (Salcher et al. 2019), and a microbiome in *Nodosilinea*, a genus of cyanobacteria widely distributed in aquatic environments (Schembri and Zammit 2022), indicates potential differences in foraging site and biofilm. Notably, a study on the gut microbiome in Tibetan wetland birds also highlighted the abundance of *Nodosilinea* in TSP (Bo et al. 2022). Despite the close genetic relationship between these species, variations in behaviour, particularly the preference on reproduction and foraging sites: KP prefer sand land, which closer to the lake coast while Tibet sand plover prefer grassland, which away from the lake (nest site location in Supplementary Figure 5), may lead to differences in diet and habits. These distinctions may contribute to the slightly differing relative abundance of specific genus microbiome in their respective guts.

When comparing females and males, in KP, we noticed the signal of enriched *Candidatus* ‘Arthomitus’ was dominantly from the female individual hinting the different food resources in two sex groups. Besides, same pattern also occurs in Acinetobacter, which regarded as a potential pathogen (Thomas et al. 2011), and a similar result is also be reported in female passerine migratory birds (Shin et al. 2019). *Staohylococcus* also exhibits a female bias, with some microbiome species in this genus discovered in the skin and internal organs of both domesticated and wild birds, demonstrating the potential to cause disease (Harry 1967, Quist et al. 2011). This result suggests the female might be exposed to higher risk of infection and more variable bacterial environment. We suspect this may stem from the high demand for nutrition for laying eggs and the discriminatory behaviour during the breeding season, which may force the female individual to make more efforts on foraging (Morrison and Hobson 2004, Halimubieke et al. 2024). Furthermore, a more male-biased genus *Catellicoccus* is also identified, this result also consists with research comparing the gut microbiome composition of two sexes in thick-billed murres (*Uria lomvia*) (Góngora et al. 2021).

Our results also showed that the difference of α-diversity and β-diversity occurs when comparing the two sexes groups in the same age. In adults, gut microbiome community of females turns to keep higher evenness instead of only considering the richness which leads a relatively stable complexity. The insight of this result would be, given that these two species are both monogamous, the mating system might fail to explain the difference in diversity between two sexes. From this, we suspect this difference might be driven by the sex hormone level (Flores et al. 2012, Shin et al. 2019, d’Afflitto et al. 2022, Yan et al. 2022). In humans, testosterone level was found to be positively correlated with the abundance of Acinetobacter in males (Shin et al. 2019), but no clear evidence proves whether hormone level will influence the abundance of this genus in avian species. Existing evidence does indicate an association between hormone levels and microbiome diversity in birds: in rufous-collared sparrows (*Z. capensis*), the diversity of gut microbiome community had a positive correlation with testosterone concentration (Dos Remedios et al. 2015). Nevertheless, this speculation needs more accurate data on the hormone level to be testified; some hormones with possibility would be estrogen (Flores et al. 2012, Fuhrman et al. 2014) and testosterone (Ridlon et al. 2013, Shin et al. 2019).

Our result of WGCNA network showed that adults and juveniles may have different diet: juveniles prefer to more proportion of vegetations in their diet, as *Exiguobacterium* is more enriched. Some species in *Exiguobacterium* have already been proven to be plant growth-promoting bacteria and help accumulate selenium (Marfetán et al. 2023). Similarly, the high proportion of vegetation in juvenile diets was also observed in Western sandpipers (*Calidris mauri*) (Hall et al. 2021). This may result from the inadequate foraging experience to acquire food with high trophic level (Durell 2003, Kozik et al. 2022). Besides, this result also implies that gut microbiome composition of shorebirds in the early stage is mainly shifted by the outside environment and diet rather than physiological factors like sex; a previous study has proved that microbial colonization in shorebird chicks starts soon after the hatching and the interaction with the environment (Grond et al. 2017).

In summary, this study described and compared the gut microbiome of KPs and TSPs. The preliminary result would be different species keep a convergence gut microbiome community but also hold several specific abundant genera due to their preference on habit. Besides, the females hold a more diverse gut microbiome environment. Finally, several microbiome phyla indicate a high proportion of vegetation on diet in juveniles. Given that the sample size of each comparison group is limited, a series of studies should be conducted. Firstly, even though we had identified several genera were enriched due to specific physiological characteristics, more functional aspects were needed study to make a solid conclusion. Secondly, the conclusion that juvenile chicks may take more plants in their diet needs more valid evidence like anatomy data and behavioural observation. Thirdly, our results did not provide a sufficient sample size to compare the diversity between males and females in juveniles nor characterize the dynamic process of gut microbiome from herbivorous diet to carnivorous diet. In consequence, in the following investigation, juvenile samples with detailed age information (specified by days) and in repeated frequency should be collected to fill this gap Finally, during the analysis, the isolation of 16S rRNA cannot portray subtle differences in the microbiome composition in these two closely related species, and more delicate sequencing method, like metagenome sequencing, should be applied in the future.

## Ethical statement

The animal study was reviewed and approved by the University of Bath Animal Welfare and Ethical Review Body (AWERB; review number: NL1905-2). All methods were carried out in accordance with relevant guidelines and regulations of the National Forestry and Grassland Administration, PRC. Birds were ringed and handled by trained people aiming to cause as little disturbance to birds as possible.

## Supplementary Material

xtae020_Supplemental_Files

## Data Availability

All data will be archived once the manuscript is accepted for publication.

## References

[bib1] Badal VD , Vaccariello ED, Murray ER et al. The gut microbiome, aging, and longevity: a systematic review. Nutrients. 2020;12:3759. 33297486 10.3390/nu12123759 PMC7762384

[bib2] Bo T , Song G, Tang S et al. Incomplete concordance between host phylogeny and gut microbial community in Tibetan wetland birds. Front Microbiol. 2022;13:848906. 35663854 10.3389/fmicb.2022.848906 PMC9161150

[bib3] Bolnick DI , Snowberg LK, Hirsch PE et al. Individual diet has sex-dependent effects on vertebrate gut microbiota. Nat Commun. 2014;5:4500. 25072318 10.1038/ncomms5500 PMC4279269

[bib4] Cao J , Hu Y, Liu F et al. Metagenomic analysis reveals the microbiome and resistome in migratory birds. Microbiome. 2020;8:26. 32122398 10.1186/s40168-019-0781-8 PMC7053137

[bib5] Caporaso JG , Kuczynski J, Stombaugh J et al. QIIME allows analysis of high-throughput community sequencing data. Nat Methods. 2010;7:335–6. 20383131 10.1038/nmeth.f.303 PMC3156573

[bib6] Caporaso JG , Lauber CL, Walters WA et al. Ultra-high-throughput microbial community analysis on the Illumina HiSeq and MiSeq platforms. ISME J. 2012;6:1621–4. 22402401 10.1038/ismej.2012.8 PMC3400413

[bib7] Cedar Lake Ventures, Inc . WeatherSpark: climate and average weather year round in Qinghaihu, China. 2023. https://weatherspark.com/countries/CN (24 May 2024, date last accessed).

[bib8] Cole JR , Wang Q, Fish JA et al. Ribosomal Database Project: data and tools for high throughput rRNA analysis. Nucl Acids Res. 2014;42:D633–42. 24288368 10.1093/nar/gkt1244 PMC3965039

[bib9] Cox NA , Richardson LJ, Maurer JJ et al. Evidence for horizontal and vertical transmission in *Campylobacter* passage from hen to her progeny. J Food Prot. 2012;75:1896–902. 23043845 10.4315/0362-028.JFP-11-322

[bib10] d'Afflitto M , Upadhyaya A, Green A et al. Association between sex hormone levels and gut microbiota composition and diversity-a systematic review. J Clin Gastroenterol. 2022;56:384–92. 35283442 10.1097/MCG.0000000000001676 PMC7612624

[bib11] Degnan PH , Pusey AE, Lonsdorf EV et al. Factors associated with the diversification of the gut microbial communities within chimpanzees from Gombe National Park. Proc Natl Acad Sci USA. 2012;109:13034–9. 22826227 10.1073/pnas.1110994109 PMC3420156

[bib12] Dixon PJJ . VEGAN, a package of R functions for community ecology. J Veg Sci. 2003;14:927–30.

[bib13] Dominianni C , Sinha R, Goedert JJ et al. Sex, body mass index, and dietary fiber intake influence the human gut microbiome. PLoS One. 2015;10:e0124599. 25874569 10.1371/journal.pone.0124599 PMC4398427

[bib14] Dominianni C , Wu J, Hayes RB et al. Comparison of methods for fecal microbiome biospecimen collection. BMC Microbiol. 2014;14:103. 24758293 10.1186/1471-2180-14-103 PMC4005852

[bib15] Dos Remedios N , Lee PL, Burke T et al. North or south? Phylogenetic and biogeographic origins of a globally distributed avian clade. Mol Phylogenet Evol. 2015;89:151–9. 25916188 10.1016/j.ympev.2015.04.010

[bib16] Durell S. The implications for conservation of age and sex-related feeding specializations in shorebirds. Wader Study Group Bull. 2003;100:35–39.

[bib17] Elderman M , de Vos P, Faas M. Role of microbiota in sexually dimorphic immunity. Front Immunol. 2018;9:1018. 29910797 10.3389/fimmu.2018.01018 PMC5992421

[bib18] Escallón C , Becker MH, Walke JB et al. Testosterone levels are positively correlated with cloacal bacterial diversity and the relative abundance of Chlamydiae in breeding male rufous-collared sparrows. Funct Ecol. 2017;31:192–203.

[bib19] Faith JJ , Guruge JL, Charbonneau M et al. The long-term stability of the human gut microbiota. Science. 2013;341:44. 10.1126/science.1237439 PMC3791589 23828941

[bib20] Flint HJ , Scott KP, Duncan SH et al. Microbial degradation of complex carbohydrates in the gut. Gut Microbes. 2012;3:289–306. 22572875 10.4161/gmic.19897 PMC3463488

[bib21] Flores R , Shi J, Fuhrman B et al. Fecal microbial determinants of fecal and systemic estrogens and estrogen metabolites: a cross-sectional study. J Transl Med. 2012;10:253. 23259758 10.1186/1479-5876-10-253 PMC3552825

[bib22] Foster JA , Rinaman L, Cryan JF. Stress & the gut-brain axis: regulation by the microbiome. Neurobiol Stress. 2017;7:124–36. 29276734 10.1016/j.ynstr.2017.03.001 PMC5736941

[bib23] Fuhrman BJ , Feigelson HS, Flores R et al. Associations of the fecal microbiome with urinary estrogens and estrogen metabolites in postmenopausal women. J Clin Endocrinol Metab. 2014;99:4632–40. 25211668 10.1210/jc.2014-2222 PMC4255131

[bib24] Góngora E , Elliott KH, Whyte L. Gut microbiome is affected by inter-sexual and inter-seasonal variation in diet for thick-billed murres (*Uria lomvia*). Sci Rep. 2021;11:1200. 33441848 10.1038/s41598-020-80557-x PMC7806582

[bib25] González-Braojos S , Vela AI, Ruiz-de-Castaneda R et al. Age-related changes in abundance of enterococci and *Enterobacteriaceae* in pied flycatcher (*Ficedula hypoleuca*) nestlings and their association with growth. J Ornithol. 2012;153:181–8.

[bib26] Goodrich JK , Waters JL, Poole AC et al. Human genetics shape the gut microbiome. Cell. 2014;159:789–99. 25417156 10.1016/j.cell.2014.09.053 PMC4255478

[bib27] Grond K , Lanctot RB, Jumpponen A et al. Recruitment and establishment of the gut microbiome in arctic shorebirds. FEMS Microbiol Ecol. 2017;93:29069418. 10.1093/femsec/fix142 29069418

[bib28] Grond K , Ryu H, Baker AJ et al. Gastro-intestinal microbiota of two migratory shorebird species during spring migration staging in Delaware Bay, USA. J Ornithol. 2014;155:969–77.

[bib29] Grond K , Sandercock BK, Jumpponen A et al. The avian gut microbiota: community, physiology and function in wild birds. J Avian Biol. 2018;49:e01788.

[bib30] Grond K , Santo Domingo JW, Lanctot RB et al. Composition and drivers of gut microbial communities in Arctic-breeding shorebirds. Front Microbiol. 2019;10:2258. 31649627 10.3389/fmicb.2019.02258 PMC6795060

[bib31] Halimubieke N , Lin X, Almalki M et al. Breeding ecology of a high-altitude shorebird in the Qinghai-Tibetan Plateau. J Ornithol. 2024;165:713–24.

[bib32] Hall LA , De La Cruz SE, Woo I et al. Age-and sex-related dietary specialization facilitate seasonal resource partitioning in a migratory shorebird. Ecol Evol. 2021;11:1866–76. 33614009 10.1002/ece3.7175 PMC7882968

[bib33] Harry EG. Some characteristics of *Staphylococcus aureus* isolated from the skin and upper respiratory tract of domesticated and wild (Feral) birds. Res Vet Sci. 1967;8:490–9. 5183295

[bib34] Hird SM , Sanchez C, Carstens BC et al. Comparative gut microbiota of 59 Neotropical bird species. Front Microbiol. 2015;6:1403. 26733954 10.3389/fmicb.2015.01403 PMC4685052

[bib35] John GK , Mullin GE. The gut microbiome and obesity. Curr Oncol Rep. 2016;18:45. 27255389 10.1007/s11912-016-0528-7

[bib36] Kaur J , Vishnu. Chapter 8–Bacterial inoculants for rhizosphere engineering: applications, current aspects, and challenges. In: Dubey RC, Kumar P (eds), Rhizosphere Engineering. Cambridge: Academic Press, 2022, 129–50.

[bib37] Kim YS , Unno T, Kim BY et al. Sex differences in gut microbiota. World J Mens Health. 2020;38:48–60. 30929328 10.5534/wjmh.190009 PMC6920072

[bib38] Knutie SA , Gotanda KM. A non-invasive method to collect fecal samples from wild birds for microbiome studies. Microb Ecol. 2018;76:851–5. 29623358 10.1007/s00248-018-1182-4

[bib39] Kohl KD , Amaya J, Passement CA et al. Unique and shared responses of the gut microbiota to prolonged fasting: a comparative study across five classes of vertebrate hosts. FEMS Microbiol Ecol. 2014;90:883–94. 25319042 10.1111/1574-6941.12442

[bib40] Kohl KD. Diversity and function of the avian gut microbiota. J Comp Physiol B. 2012;182:591–602. 22246239 10.1007/s00360-012-0645-z

[bib41] Kozik R , Meissner W, Listewnik B et al. Differences in foraging behaviour of a migrating shorebird at stopover sites on regulated and unregulated sections of a large European lowland river. J Ornithol. 2022;163:791–802.

[bib42] Lauber CL , Zhou N, Gordon JI et al. Effect of storage conditions on the assessment of bacterial community structure in soil and human-associated samples. FEMS Microbiol Lett. 2010;307:80–86. 20412303 10.1111/j.1574-6968.2010.01965.x PMC3148093

[bib43] Laviad-Shitrit S , Izhaki I, Lalzar M et al. Comparative analysis of intestine microbiota of four wild waterbird species. Front Microbiol. 2019;10:1911. 31481943 10.3389/fmicb.2019.01911 PMC6711360

[bib44] Lewis WB , Moore FR, Wang S. Changes in gut microbiota of migratory passerines during stopover after crossing an ecological barrier. The Auk. 2016;134:137–45.

[bib45] Ley RE , Hamady M, Lozupone C et al. Evolution of mammals and their gut microbes. Science. 2008;320:1647–51. 18497261 10.1126/science.1155725 PMC2649005

[bib46] Li T , Long M, Gatesoupe F-J et al. Comparative analysis of the intestinal bacterial communities in different species of carp by pyrosequencing. Microb Ecol. 2015;69:25–36. 25145494 10.1007/s00248-014-0480-8

[bib47] Liu C , Cui Y, Li X& et al. microeco: an R package for data mining in microbial community ecology. FEMS Microbiol Ecol. 2021;97:fiaa255. 33332530 10.1093/femsec/fiaa255

[bib48] Marcelino VR , Wille M, Hurt AC et al. Meta-transcriptomics reveals a diverse antibiotic resistance gene pool in avian microbiomes. BMC Biol. 2019;17:31. 30961590 10.1186/s12915-019-0649-1 PMC6454771

[bib49] Marfetán JA , Gallo AL, Farias ME et al. *Exiguobacterium* sp. as a bioinoculant for plant-growth promotion and Selenium biofortification strategies in horticultural plants. World J Microbiol Biotechnol. 2023;39:134. 36961610 10.1007/s11274-023-03571-x

[bib50] McDonald D , Hyde E, Debelius JW et al. American gut: an open platform for citizen science microbiome research. mSystems. 2018;3:e00031–18. 29795809 10.1128/mSystems.00031-18 PMC5954204

[bib51] Mendoza MLZ , Roggenbuck M, Vargas KM et al. Protective role of the vulture facial skin and gut microbiomes aid adaptation to scavenging. Acta Vet Scand. 2018;60:61. 30309375 10.1186/s13028-018-0415-3 PMC6182802

[bib52] Michl SC , Ratten JM, Beyer M et al. The malleable gut microbiome of juvenile rainbow trout (*Oncorhynchus mykiss*): diet-dependent shifts of bacterial community structures. PLoS One. 2017;12:e0177735. 28498878 10.1371/journal.pone.0177735 PMC5428975

[bib53] Moeller AH , Peeters M, Ndjango JB et al. Sympatric chimpanzees and gorillas harbor convergent gut microbial communities. Genome Res. 2013;23:1715–20. 23804402 10.1101/gr.154773.113 PMC3787267

[bib54] Morrison RIG , Hobson KA. Use of body stores in shorebirds after arrival on high-Arctic breeding grounds. The Auk. 2004;121:333–44.

[bib55] Murray MH , Lankau EW, Kidd AD et al. Gut microbiome shifts with urbanization and potentially facilitates a zoonotic pathogen in a wading bird. PLoS One. 2020;15:e0220926. 32134945 10.1371/journal.pone.0220926 PMC7058277

[bib56] Neuman H , Debelius JW, Knight R et al. Microbial endocrinology: the interplay between the microbiota and the endocrine system. FEMS Microbiol Rev. 2015;39:509–21. 25701044 10.1093/femsre/fuu010

[bib57] Nuriel-Ohayon M , Neuman H, Koren O. Microbial changes during pregnancy, birth, and infancy. Front Microbiol. 2016;7:1031. 27471494 10.3389/fmicb.2016.01031 PMC4943946

[bib58] Org E , Mehrabian M, Parks BW et al. Sex differences and hormonal effects on gut microbiota composition in mice. Gut Microbes. 2016;7:313–22. 27355107 10.1080/19490976.2016.1203502 PMC4988450

[bib59] Pigot AL , Sheard C, Miller ET et al. Macroevolutionary convergence connects morphological form to ecological function in birds. Nat Ecol Evol. 2020;4:230–9. 31932703 10.1038/s41559-019-1070-4

[bib60] Prince AL , Pace RM, Dean T et al. The development and ecology of the Japanese macaque gut microbiome from weaning to early adolescence in association with diet. Am J Primatol. 2019;81:e22980. 31066111 10.1002/ajp.22980 PMC7020817

[bib61] Pruesse E , Quast C, Knittel K et al. SILVA: a comprehensive online resource for quality checked and aligned ribosomal RNA sequence data compatible with ARB. Nucleic Acids Res. 2007;35:7188–96. 17947321 10.1093/nar/gkm864 PMC2175337

[bib62] Que PJ , Szekely T, Wang PC et al. Offspring sex ratio is unrelated to parental quality and time of breeding in a multiple-breeding shorebird. J Ornithol. 2019;160:443–52.

[bib63] Quist EM , Belcher C, Levine G et al. Disseminated histoplasmosis with concurrent oral candidiasis in an Eclectus parrot (*Eclectus roratus*). Avian Pathol. 2011;40:207–11. 21500041 10.1080/03079457.2011.554796

[bib64] Ridlon JM , Ikegawa S, Alves JM et al. *Clostridium scindens*: a human gut microbe with a high potential to convert glucocorticoids into androgens. J Lipid Res. 2013;54:2437–49. 23772041 10.1194/jlr.M038869 PMC3735941

[bib65] Risely A , Waite DW, Ujvari B et al. Active migration is associated with specific and consistent changes to gut microbiota in *Calidris* shorebirds. J Anim Ecol. 2018;87:428–37. 29111601 10.1111/1365-2656.12784

[bib66] Ritchie ME , Phipson B, Wu D et al. limma powers differential expression analyses for RNA-sequencing and microarray studies. Nucleic Acids Res. 2015;43:e47–. 25605792 10.1093/nar/gkv007 PMC4402510

[bib67] Roggenbuck M , Schnell IB, Blom N et al. The microbiome of New World vultures. Nat Commun. 2014;5:5498. 25423494 10.1038/ncomms6498

[bib68] Rognes T , Flouri T, Nichols B et al. VSEARCH: a versatile open source tool for metagenomics. PeerJ. 2016;4:e2584. 27781170 10.7717/peerj.2584 PMC5075697

[bib69] Rothschild D , Weissbrod O, Barkan E et al. Environment dominates over host genetics in shaping human gut microbiota. Nature. 2018;555:210–5. 29489753 10.1038/nature25973

[bib70] Ryu H , Griffith JF, Khan IU et al. Comparison of gull feces-specific assays targeting the 16S rRNA genes of *Catellicoccus marimammalium* and *Streptococcus* spp. Appl Environ Microb. 2012;78:1909–16. 10.1128/AEM.07192-11 PMC3298170 22226950

[bib71] Salcher MM , Schaefle D, Kaspar M et al. Evolution in action: habitat transition from sediment to the pelagial leads to genome streamlining in Methylophilaceae. ISME J. 2019;13:2764–77. 31292537 10.1038/s41396-019-0471-3 PMC6794327

[bib72] Santos SS , Pardal S, Proença DN et al. Diversity of cloacal microbial community in migratory shorebirds that use the Tagus estuary as stopover habitat and their potential to harbor and disperse pathogenic microorganisms. FEMS Microbiol Ecol. 2012;82:63–74. 22571242 10.1111/j.1574-6941.2012.01407.x

[bib73] Schembri S , Zammit G. The biodiversity of epilithic microalgal communities colonising a central Mediterranean coastline. J Coast Res. 2022;38:249–60.

[bib74] Shi P , Chen Y, Zhang G et al. Factors contributing to spatial–temporal variations of observed oxygen concentration over the Qinghai-Tibetan Plateau. Sci Rep. 2021;11:17338. 34462465 10.1038/s41598-021-96741-6 PMC8405649

[bib75] Shin JH , Park YH, Sim M et al. Serum level of sex steroid hormone is associated with diversity and profiles of human gut microbiome. Res Microbiol. 2019;170:192–201. 30940469 10.1016/j.resmic.2019.03.003

[bib76] Sinigalliano CD , Ervin JS, Van De Werfhorst LC et al. Multi-laboratory evaluations of the performance of *Catellicoccus marimammalium* PCR assays developed to target gull fecal sources. Water Res. 2013;47:6883–96. 23916157 10.1016/j.watres.2013.02.059

[bib77] Smith OM , Snyder WE, Owen JP. Are we overestimating risk of enteric pathogen spillover from wild birds to humans?. Biol Rev. 2020;95:652–79. 32003106 10.1111/brv.12581 PMC7317827

[bib78] Song SJ , Amir A, Metcalf JL et al. Preservation methods differ in fecal microbiome stability, affecting suitability for field studies. mSystems. 2016;1:e00021–16. 10.1128/mSystems.00021-16 PMC5069758 27822526

[bib79] Song SJ , Lauber C, Costello EK et al. Cohabiting family members share microbiota with one another and with their dogs. eLife. 2013;2:e00458. 23599893 10.7554/eLife.00458 PMC3628085

[bib80] Song Z , Lin X, Que P et al. The allocation between egg size and clutch size depends on local nest survival rate in a mean of bet-hedging in a shorebird. Avian Res. 2020;11:1–10.

[bib81] Su T , Lin X, Huang Q et al. Mercury exposure in sedentary and migratory *Charadrius* plovers distributed widely across China. Environ Sci Pollut Res. 2020;27:4236–45. 10.1007/s11356-019-06873-6 31828699

[bib82] Székely T , Kosztolányi A, Küpper C. Practical Guide for Investigating Breeding Ecology of Kentish Plover Charadrius alexandrinus. Bath: University of Bath, 2008. https://www.pennuti.net/wp-content/uploads/2010/08/KP_Field_Guide_v3.pdf (24 May 2024, date last accessed).

[bib83] Székely T. Why study plovers? The significance of non-model organisms in avian ecology, behaviour and evolution. J Ornithol. 2019;160:923–33.

[bib84] Teyssier A , Matthysen E, Hudin NS et al. Diet contributes to urban-induced alterations in gut microbiota: experimental evidence from a wild passerine. Proc Biol Sci. 2020;287:20192182. 32019440 10.1098/rspb.2019.2182 PMC7031670

[bib85] Thomas F , Hehemann JH, Rebuffet E et al. Environmental and gut bacteroidetes: the food connection. Front Microbio. 2011;2:93. 10.3389/fmicb.2011.00093 PMC3129010 21747801

[bib86] Thompson CL , Vier R, Mikaelyan A et al. ‘*Candidatus* Arthromitus’ revised: segmented filamentous bacteria in arthropod guts are members of Lachnospiraceae. Environ Microbiol. 2012;14:1454–65. 22436008 10.1111/j.1462-2920.2012.02731.x

[bib87] Unterseher M , Jumpponen A, Opik M et al. Species abundance distributions and richness estimations in fungal metagenomics–lessons learned from community ecology. Mol Ecol. 2011;20:275–85. 21155911 10.1111/j.1365-294X.2010.04948.x

[bib88] van Dongen WFD , White J, Brandl HB et al. Age-related differences in the cloacal microbiota of a wild bird species. BMC Ecol. 2013;13:11. 23531085 10.1186/1472-6785-13-11 PMC3668179

[bib89] Videvall E , Strandh M, Engelbrecht A et al. Measuring the gut microbiome in birds: comparison of faecal and cloacal sampling. Mol Ecol Resour. 2018;18:424–34. 29205893 10.1111/1755-0998.12744

[bib90] Waite DW , Taylor MW. Characterizing the avian gut microbiota: membership, driving influences, and potential function. Front Microbiol. 2014;5:223. 24904538 10.3389/fmicb.2014.00223 PMC4032936

[bib91] Wang A , Zhang Z, Ding Q et al. Intestinal *Cetobacterium* and acetate modify glucose homeostasis via parasympathetic activation in zebrafish. Gut Microbes. 2021;13:1–15. 10.1080/19490976.2021.1900996 PMC8043178 33840371

[bib92] Watson SE , Hauffe HC, Bull MJ et al. Global change-driven use of onshore habitat impacts polar bear faecal microbiota. ISME J. 2019;13:2916–26. 31378786 10.1038/s41396-019-0480-2 PMC6864082

[bib93] Wei C , Schweizer M, Tomkovich PS et al. Genome-wide data reveal paraphyly in the sand plover complex (*Charadrius mongolus*/*leschenaultii*). Ornithology. 2022;139:ukab085.

[bib94] Wu D , Wang C, Simujide H et al. Reproductive hormones mediate intestinal microbiota shifts during estrus synchronization in grazing Simmental cows. Animals. 2022;12:1751. 35883298 10.3390/ani12141751 PMC9311722

[bib95] Xu W , Xu N, Zhang Q et al. Association between diet and the gut microbiome of young captive red-crowned cranes (*Grus japonensis*). BMC Vet Res. 2023;19:80. 37391732 10.1186/s12917-023-03636-x PMC10311889

[bib96] Yan R , Lu M, Zhang L et al. Effect of sex on the gut microbiota characteristics of passerine migratory birds. Front Microbiol. 2022;13:917373. 36118231 10.3389/fmicb.2022.917373 PMC9478027

[bib97] Yan W , Sun CJ, Zheng JX et al. Efficacy of fecal sampling as a gut proxy in the study of chicken gut microbiota. Front Microbiol. 2019;10:2126. 31572332 10.3389/fmicb.2019.02126 PMC6753641

[bib98] Zhang H , Zhang Z, Liao Y et al. The complex link and disease between the gut microbiome and the immune system in infants. Front Cell Infect Microbiol. 2022;12:924119. 35782111 10.3389/fcimb.2022.924119 PMC9241338

[bib99] Zhang M , Chen H, Liu L et al. The changes in the frog gut microbiome and its putative oxygen-related phenotypes accompanying the development of gastrointestinal complexity and dietary shift. Front Microbiol. 2020;11:162. 32194513 10.3389/fmicb.2020.00162 PMC7062639

[bib100] Zhu Y , Li Y, Yang H et al. Establishment of gut microbiome during early life and its relationship with growth in endangered crested ibis (*Nipponia nippon*). Front Microbiol. 2021;12:723682. 34434183 10.3389/fmicb.2021.723682 PMC8382091

