# Supplementary material for: Gut microbiome in two high-altitude bird populations showed heterogeneity in sex and life stage
Source: FEMS Microbes. 2024 Jul 4;5:xtae020. doi: 10.1093/femsmc/xtae020 (PMC11462087; doi:10.1093/femsmc/xtae020)
Supplement: xtae020_Supplemental_Files [file xtae020_supplemental_files.zip › FEMSMC-2024-008.R2 one sentence summary.docx]

Using 16s rRNA characterize the gut microbiome community in two plover species in high-altitude population to show difference on sex and ages.
