# Supplementary material for: Gut microbiome in two high-altitude bird populations showed heterogeneity in sex and life stage
Source: FEMS Microbes. 2024 Jul 4;5:xtae020. doi: 10.1093/femsmc/xtae020 (PMC11462087; doi:10.1093/femsmc/xtae020)
Supplement: xtae020_Supplemental_Files [file xtae020_supplemental_files.zip › Supplementary file1_figure_table.docx]

***Supplementary Material***

**1 Supplementary Tables and Figures**

**1.1 Supplementary Table**

**Supplementary Table 1.** Quality status of 16s rRNA library each sample

| **Sample** | **Length(bp)** | **Reads** | **Bases(bp)** | **Q20(%)** | **Q30(%)** | **GC(%)** |
| --- | --- | --- | --- | --- | --- | --- |
| D346712 | 250 | 127250 | 31812500 | 97.91 | 93.99 | 56.44 |
| D346773 | 250 | 201550 | 50387500 | 97.84 | 93.64 | 53.58 |
| D346774 | 250 | 188534 | 47133500 | 93.59 | 90.74 | 48.39 |
| D346775 | 250 | 162746 | 40686500 | 97.55 | 93.28 | 53.57 |
| D346776 | 250 | 187692 | 46923000 | 94.18 | 91.31 | 51.58 |
| D346777 | 250 | 154212 | 38553000 | 97.4 | 92.55 | 52.05 |
| D346778 | 250 | 213468 | 53367000 | 95.6 | 93.41 | 51.02 |
| D346779 | 250 | 187420 | 46855000 | 97.63 | 93.36 | 52.97 |
| D346781 | 250 | 97296 | 24324000 | 95.75 | 89.27 | 52.68 |
| D346782 | 250 | 196032 | 49008000 | 94.41 | 91.69 | 53.97 |
| D346785 | 250 | 230596 | 57649000 | 97.57 | 93.14 | 51.71 |
| D346786 | 250 | 151246 | 37811500 | 94.52 | 92.05 | 47.69 |
| D346789 | 250 | 193568 | 48392000 | 96.08 | 89.67 | 50.44 |
| D346790 | 250 | 183240 | 45810000 | 97.59 | 93.37 | 54.02 |
| D346792 | 250 | 197078 | 49269500 | 97.65 | 93.33 | 53.92 |
| D346793 | 250 | 186366 | 46591500 | 97.57 | 93.2 | 52.73 |
| D346798 | 250 | 155112 | 38778000 | 96.66 | 94.97 | 53.24 |
| D346801 | 250 | 188228 | 47057000 | 97.44 | 92.96 | 53.1 |
| D346802 | 250 | 153438 | 38359500 | 95.87 | 89.37 | 52.52 |
| D346803 | 250 | 211438 | 52859500 | 97.53 | 92.96 | 50.87 |
| D346807 | 250 | 172176 | 43044000 | 97.58 | 93.31 | 56.54 |
| D346809 | 250 | 173870 | 43467500 | 97.75 | 93.46 | 51.61 |
| D346811 | 250 | 201256 | 50314000 | 97.09 | 92.78 | 56.42 |
| D346814 | 250 | 176436 | 44109000 | 97.6 | 93.47 | 55.74 |
| D346819 | 250 | 219356 | 54839000 | 97.35 | 92.43 | 53.44 |
| D346821 | 250 | 170916 | 42729000 | 95.84 | 89.23 | 52.79 |
| D346822 | 250 | 141056 | 35264000 | 94.21 | 91.34 | 53.3 |
| D346823 | 250 | 130366 | 32591500 | 96.59 | 90.63 | 53.49 |
| D346824 | 250 | 141780 | 35445000 | 97.74 | 93.54 | 53.44 |
| D346825 | 250 | 262586 | 65646500 | 97.71 | 93.41 | 51.06 |
| D346827 | 250 | 122204 | 30551000 | 97.34 | 92.45 | 53.2 |
| D346828 | 250 | 204694 | 51173500 | 97.32 | 92.42 | 53.69 |
| D346829 | 250 | 234196 | 58549000 | 97 | 91.71 | 52.83 |

**Supplementary Table 2.** Result of Chao OTU amounts in sex and age groups in different species

| **Groups** | **KP** | **TSP** |
| --- | --- | --- |
| **Female unique** | 2015 | 547 |
| **Male unique** | 628 | 320 |
| **Female Male shared** | 1205 | 512 |
| **Adult unique** | 2423 | 577 |
| **Juvenile unique** | 280 | 460 |
| **Adult Juvenile shared** | 1145 | 369 |

**Supplementary Table 3.** Alpha diversity of each sample under rarefaction

| Sample | ACE | Chao1 | Shannon | Simpson | Goods_Coverage |
| --- | --- | --- | --- | --- | --- |
| D346712 | 282.672 | 290.091 | 3.871 | 0.686 | 0.999 |
| D346776 | 296.489 | 308 | 5.41 | 0.949 | 0.999 |
| D346777 | 301.766 | 306 | 4.21 | 0.882 | 0.999 |
| D346778 | 225.402 | 229.667 | 4.884 | 0.91 | 0.999 |
| D346827 | 361.178 | 384.75 | 3.861 | 0.782 | 0.998 |
| D346829 | 355.218 | 359.774 | 2.892 | 0.684 | 0.998 |
| D346822 | 338.595 | 349.333 | 2.156 | 0.437 | 0.999 |
| D346823 | 813.919 | 777 | 2.052 | 0.389 | 0.995 |
| D346828 | 651.779 | 631.679 | 5.021 | 0.932 | 0.996 |
| D346775 | 294.322 | 306 | 3.449 | 0.624 | 0.999 |
| D346779 | 252.648 | 294 | 3.05 | 0.603 | 0.999 |
| D346781 | 60.157 | 57.143 | 3.409 | 0.83 | 1 |
| D346782 | 578.811 | 597.875 | 6.467 | 0.97 | 0.999 |
| D346786 | 583.377 | 573.679 | 2.777 | 0.555 | 0.997 |
| D346789 | 696.07 | 720.386 | 5.334 | 0.903 | 0.996 |
| D346801 | 329.328 | 343.214 | 5.458 | 0.926 | 0.999 |
| D346807 | 1821.605 | 1823.415 | 8.983 | 0.992 | 0.997 |
| D346814 | 413.427 | 421.875 | 7.239 | 0.981 | 0.999 |
| D346824 | 261.121 | 258.324 | 2.201 | 0.537 | 0.999 |
| D346792 | 1098.484 | 1084.879 | 4.268 | 0.746 | 0.994 |
| D346793 | 209.903 | 209.333 | 3.541 | 0.756 | 0.999 |
| D346798 | 463.479 | 435.02 | 0.872 | 0.175 | 0.997 |
| D346802 | 278.522 | 266.6 | 2.072 | 0.447 | 0.998 |
| D346803 | 444.141 | 448.692 | 4.617 | 0.901 | 0.998 |
| D346785 | 622.746 | 592.054 | 2.776 | 0.691 | 0.996 |
| D346819 | 689.299 | 699.109 | 1.992 | 0.351 | 0.996 |
| D346821 | 629.251 | 622.088 | 2.616 | 0.546 | 0.996 |
| D346774 | 701.624 | 709.171 | 3.373 | 0.618 | 0.997 |
| D346825 | 444.339 | 479.531 | 4.569 | 0.898 | 0.998 |
| D346773 | 626.007 | 624.3 | 1.428 | 0.284 | 0.996 |
| D346790 | 408.694 | 425.207 | 4.461 | 0.885 | 0.999 |
| D346809 | 544.554 | 561.938 | 3.535 | 0.76 | 0.997 |
| D346811 | 276.991 | 274.5 | 3.751 | 0.826 | 0.999 |

**Supplementary Table 4.** Result of Chao1 index, Shannon index and Simpson index of faecal samples of hosts of different species, sex and age groups using ANOVA.

| **Groups** | **Chao1** | | **Shannon** | **Simpson** |
| --- | --- | --- | --- | --- |
| **Species** | F=1.002, p=0.326 | *F=0.002, p=0.9675* | | *F=0.23, p=0.636* |
| **Sex** | *F=0.038, p=0.847* | ***F=4.499, p=0.044*** | | *F=2.088, p=0.9675* |
| **Age** | *F=0.527, p=0.474* | *F=0.149, p=0.9675* | | *F=0.009, p=0.1609* |
| **Species*Sex** | *F=0.002, p=0.651* | *F=0.255, p=0.6182* | | *F=0.034, p=0.8554* |
| **Species*Age** | *F=1.458, p=0.239* | *F=0, p=0.9888* | | *F=0.292, p=0.5938* |
| **Sex*Age** | *F=0.46, p=0.504* | ***F=5.451, p=0.0279*** | | ***F=7.685, p=0.0104*** |
| **Species*Age*Sex** | *F=0, p=0.988* | *F=0.604, p=0.4444* | | *F=1.381, p=0.2509* |

**Supplementary Table 5.** Result of 29 PCoA components of Bray-Curtis distance comparing different species, sexes and age groups using PERMANOVA.

| **Groups** | **F** | | **R^2^** | **P-value** |
| --- | --- | --- | --- | --- |
| **Species** | -71.993 | *-0.222* | | *0.941* |
| **Sex** | *-276.100* | *-0.851* | | *0.995* |
| **Age** | *277.681* | *0.856* | | ***0.022*** |
| **Species*Sex** | *-66.359* | *-0.205* | | *0.933* |
| **Species*Age** | *83.883* | *0.259* | | *0.092* |
| **Sex*Age** | *265.701* | *0.820* | | ***0.019*** |
| **Species*Age*Sex** | *86.319* | *0.266* | | *0.073* |

**Supplementary Table 6.** Differential expression analysis of 12 genus in module green in different age group using limma.

| **Genus** | **Log2FC** | | **P-value** | **Q-value** |
| --- | --- | --- | --- | --- |
| **Exiguobacterium** | **0.334** | ***0.0026*** | | ***0.032*** |
| **Escherichia-Shigella** | *3.027* | *0.033* | | *0.170* |
| **Enterococcus** | *2.548* | *0.054* | | *0.170* |
| **Luteolibacter** | *0.117* | *0.057* | | *0.170* |
| **[Clostridium]_innocuum_group** | *0.801* | *0.099* | | *0.192* |
| **Arsenophonus** | *0.123* | *0.106* | | *0.192* |
| **f__Rhizobiaceae_Unclassified** | *1.762* | *0.112* | | *0.192* |
| **Allorhizobium-Neorhizobium-Pararhizobium-Rhizobium** | *0.233* | *0.169* | | *0.253* |
| **Brevundimonas** | *0.054* | *0.248* | | *0.330* |
| **Burkholderia-Caballeronia-Paraburkholderia** | *0.042* | *0.294* | | *0.352* |
| **Lactobacillus** | *0.265* | *0.341* | | *0.371* |
| **Leifsonia** | *0.123* | *0.548* | | *0.547* |

**1.2 Supplementary Figures**

**Rarefaction curve (Supplementary Figure 1)**

**Comparison of alpha diversity in age and sex group (Supplementary Figure 2)**

**Comparison of alpha diversity between sex in same age group using Kentish Plover sample (Supplementary Figure 3)**

**PcoA plot using all OTUs (Supplementary Figure 4)**

**Nest distribution near Qinghai Lake (Supplementary Figure 5)**


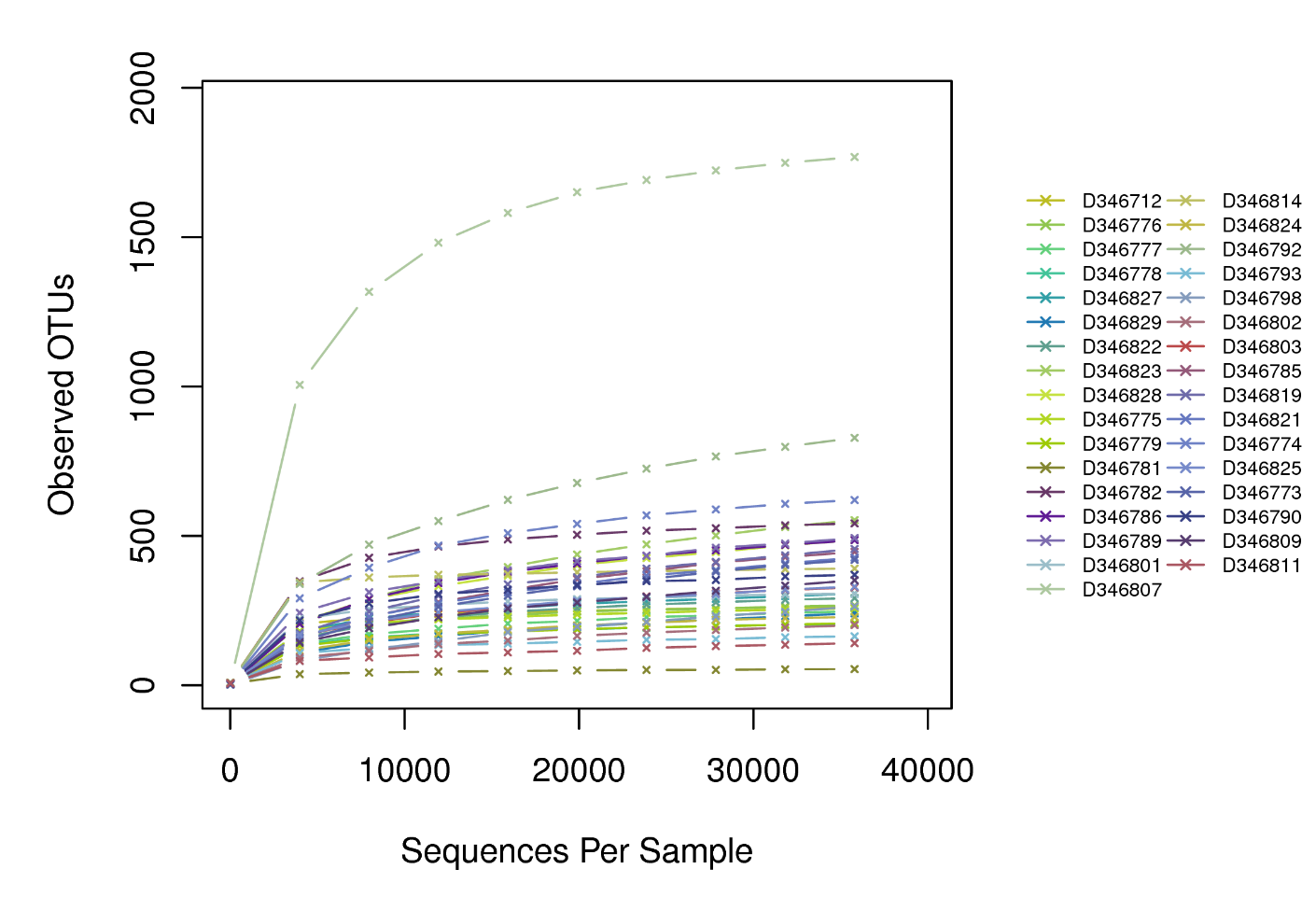


**Supplementary Figure 1.** Rarefaction abundance curve of 33 samples


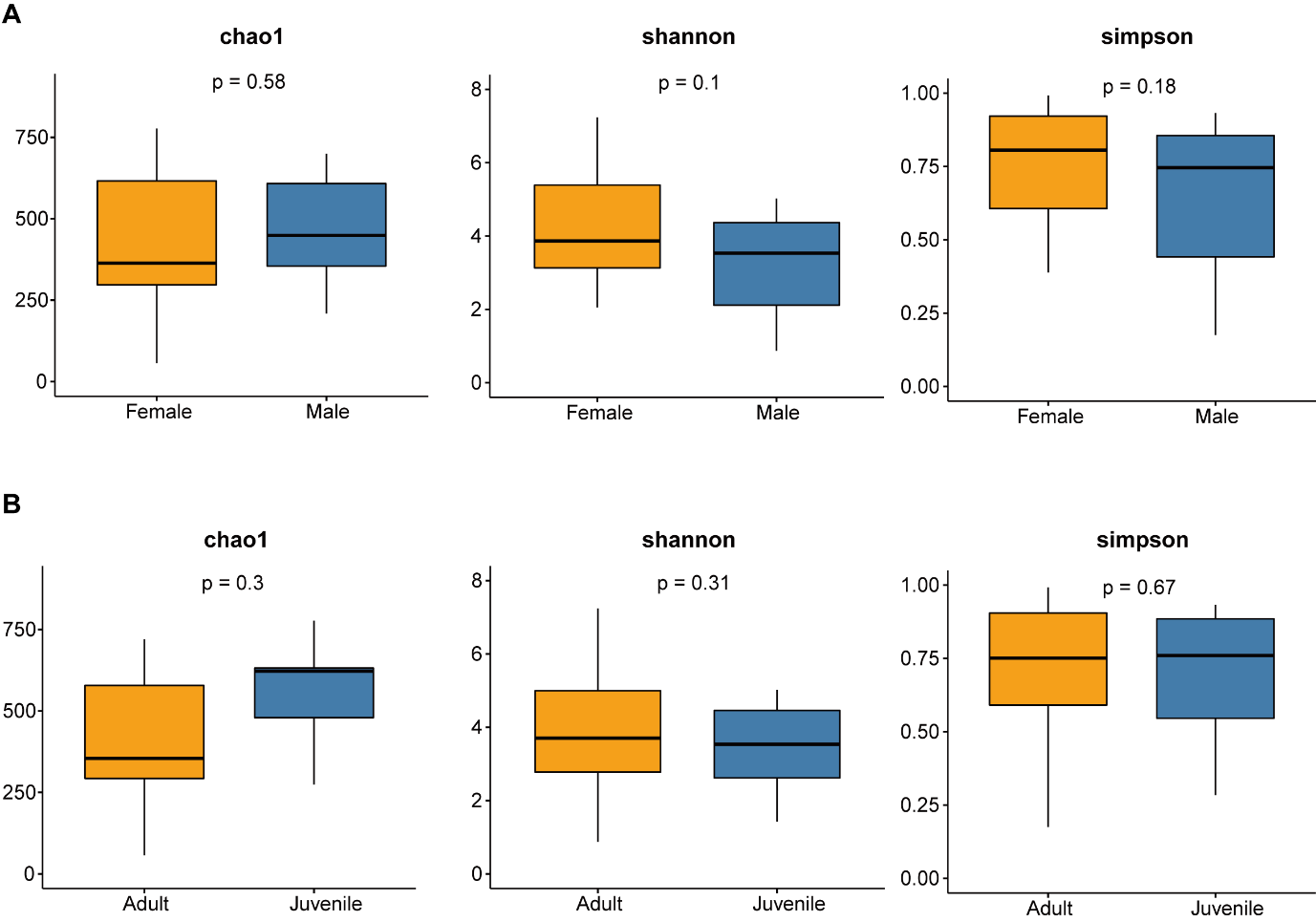


**Supplementary Figure 2.** Comparison alpha diversity in sex **(A)** and age **(B)**group


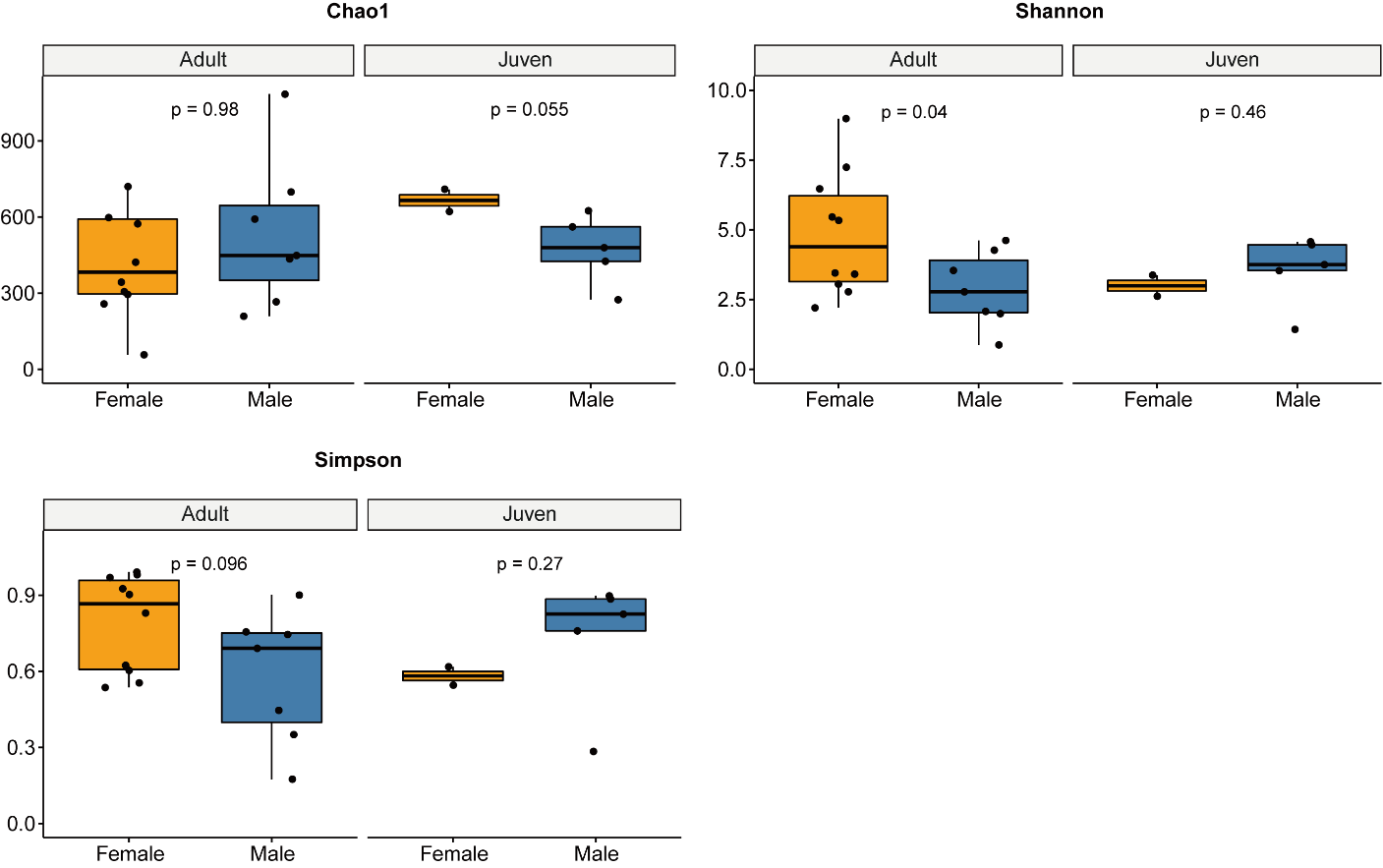


**Supplementary Figure 3.** Comparison alpha diversity between sex in same age group using Kentish plover samples


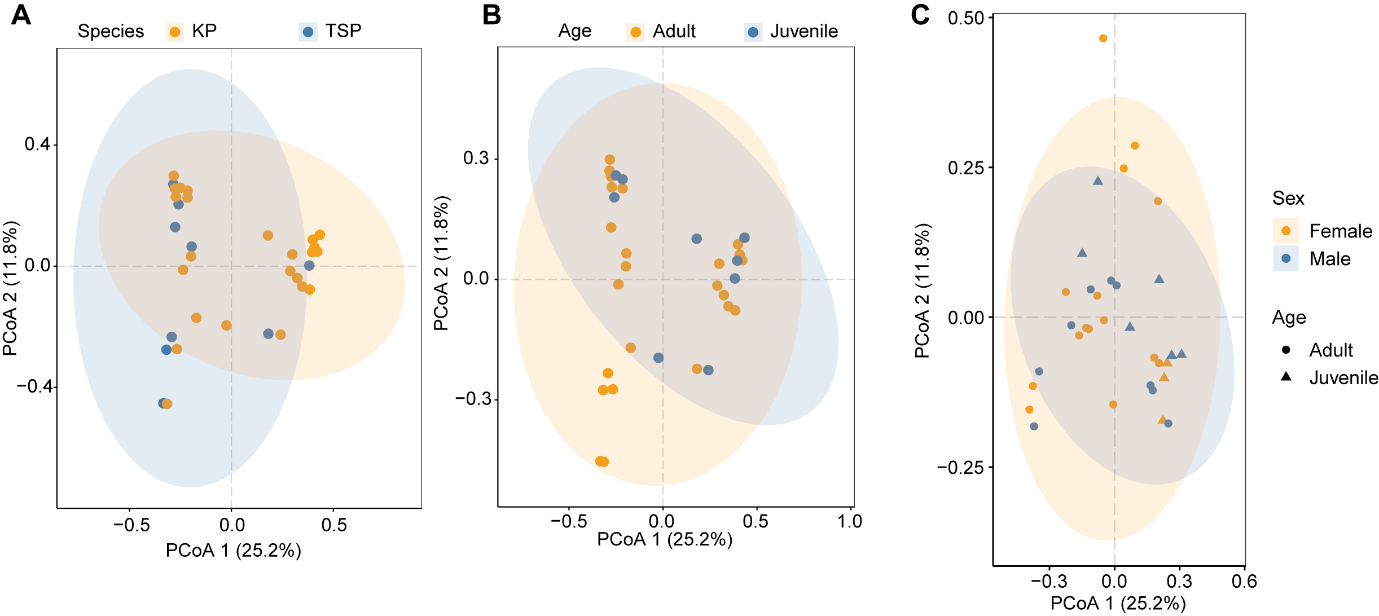


**Supplementary Figure 4.** Principal coordinate analyses (PCoA) based on Bray-Curtis distance matrices using all OTUs showing the differences in microbial composition between **(A)** species, **(B)** sex, **(C)** age group


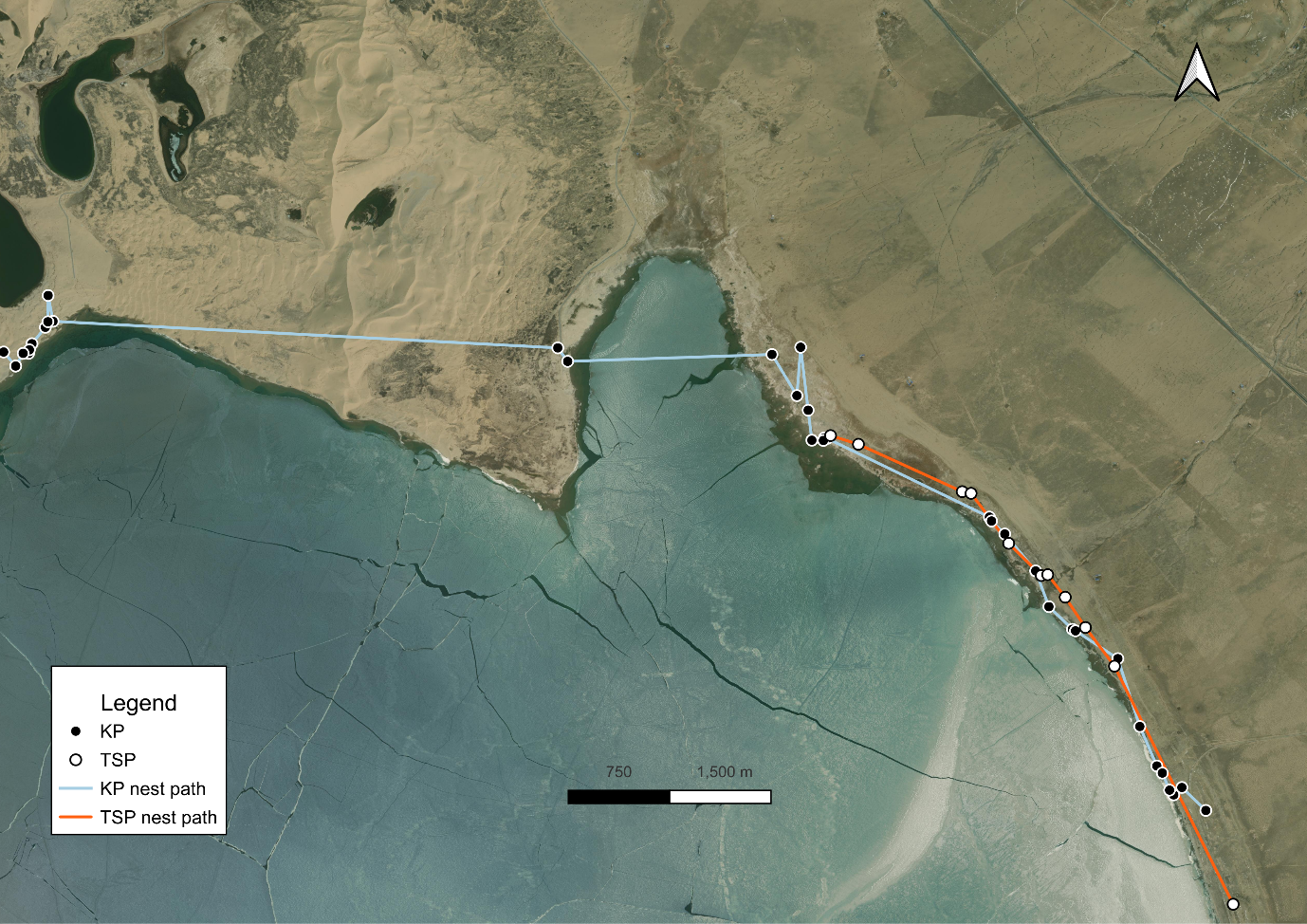


**Supplementary Figure 5.** Nest distribution of Kentish plover and Tibetan sand plover in 2023, nest path is linked according the descending order of longitude. Figure was plot by QGIS (ver 3.36.1) using the latest ESRI satellite map ([https://server.arcgisonline.com/ArcGIS/rest/services/World_Imagery/MapServer/tile/{z}/{y}/{x}](https://server.arcgisonline.com/ArcGIS/rest/services/World_Imagery/MapServer/tile/%7Bz%7D/%7By%7D/%7Bx%7D))
