# Supplementary material for: Gut microbiome in two high-altitude bird populations showed heterogeneity in sex and life stage
Source: FEMS Microbes. 2024 Jul 4;5:xtae020. doi: 10.1093/femsmc/xtae020 (PMC11462087; doi:10.1093/femsmc/xtae020)
Supplement: xtae020_Supplemental_Files [file xtae020_supplemental_files.zip › FEMSMC-2024-008.r2 supplementary data - reviews and author response.docx]

**Reviews of manuscript FEMSMC-2024-008**

**Reviewer 1**

**Recommendation: Major Revision**

In this paper the authors aimed to understand how host species, sex, and age influence the gut microbiome of two plover species. Whilst they identified few differences amongst species, they identified a sex by age interaction effect. This topic is of interest but not particularly novel- plenty of papers have investigated these questions in bird species and have utilised far greater sample sizes than presented here. I also have several methodological/statistical concerns that would need to be addressed prior to publication.

Major comments

1. It is unclear to me how the results have been analysed. The authors state (line 171) that faecal samples were classified into three groups- Kentish plover (KP) vs Sand plover (SP); female KP vs male KP; adult KP vs juvenile KP. However, it is not clear from the methods, results, Figures or Table legends whether separate analyses were carried out for each of these groups in turn or in some other way. Further to this, the authors have extremely low sample sizes, both for sand plovers (just 1 sample each for male and female juveniles and not much better for adults) and for Kentish plovers (e.g. only 2 juvenile females). I don’t believe they have the power to test for an age*sex interaction at all. A common rule of thumb in statistics is that you should have 10 data points for each predictor term you include in your model; as there are only 33 samples in total (and 24 for KP if analysing on its own) this means at most you could include 2-3 terms in the model before it becomes over-parameterised. Including too many terms may give erroneous results.

In light of this, I would suggest the authors simplify their analyses. Perhaps they could first run an analysis using adult KP and adult SP to see if there are differences across species (using species as a predictor whilst also controlling for sex in the same model). Then use KP samples to test for the effects of age and sex in a second model. These two analyses should be done for alpha and adonis analyses.

2. How did the authors control for differences in sequencing depth across samples? A common practice would be to rarefy reads (i.e. normalise to same read depth) prior to alpha diversity calculations. Otherwise, libraries with greater numbers of reads could generate greater diversity scores. This would be a problem if read depth is confounded with a variable of interest (e.g. if SP samples consistently have lower library sizes than KP, or juveniles have lower read depth than adults). I refer them to this recent publication for an in depth explanation of this: https://journals.asm.org/doi/10.1128/msphere.00355-23 . I would suggest that the authors check whether their results are consistent when they rarefy reads.

Minor comments:

1. Line 41-42: The results presented here are not strongly linked to conservation. I would remove the final part of this sentence alluding to that.

2. The second paragraph of the introduction is extremely long. I would suggest splitting into new paragraphs 1) when sex is introduced as a factor (line 71), 2) when development is introduced (line 87), 3) when study species is introduced (line 103)

3. Line 82- mice and cows don’t have cloacas. Do you mean the vaginal microbiome? Or reproductive tract microbiome?

4. Line 95- incomplete maturation of the host immune system or gut morphology in juveniles could also be a factor leading to differences between juvenile and adult microbiota.

5. Were any controls collected at the time of sampling (e.g. from collectors hands, empty faecal bags etc..). Also, were any negative extraction or PCR blanks included in the sequencing run? These controls are very important in microbiome studies where contaminants could be introduced during sample collection or from laboratory agents leading to elevated diversity and spurious differences across samples.

6. Line 188 and 201- OUT should be OTU

7. Line 189- “cloacal swabs” should be changed to “faecal samples” as these imply different sample types. I believe you collected the latter

8. Figure 1- perhaps consider labelling adult/juvenile and male/female samples in the plot for clarity (e.g. by ordering bars and adding facets).

9. Figure 1 c-e How were the taxa selected for these heatmaps/comparisons? Also, lines 206-226 (particularly line 215 “no significant variance between sexes”): heatmaps are not an appropriate method to statistically compare abundances of bacteria between samples and should only be used for visualisation following a statistical differential abundance test e.g. an ANCOM analysis.

10. Line 222- was the core microbiome calculated across both species combined, or for KP and SP separately? Differences in sample size would mean the latter is most appropriate. Please clarify in the text.

11. Figures 3 & S2- please add all raw data points to box plots to show the spread of the data- particularly important as you have low sample sizes.

12. Figure 4- Why did the authors only include taxa with an LDA of >2 (from LEFSE analysis) in this plot? That would give the appearance of greater differentiation than there actually is across samples. Also where are the results of the LEFSE analysis reported (this is linked to my point 9 above- what differential abundance tests were carried out)?

13. Line 237- incorrect figure referenced- change FigS1 to FigS2.

14. Line 250- incorrect figure referenced- change FigS2 to FigS3?

**Reviewer 2: Zhengwang Zhang**

**Recommendation: Minor Revision**

Gut microbiotas affects the reproductive success and survival of birds.In this manuscript, the authors collected samples from two breeding populations of Kentish plover (Charadrius alexandrinus) and Tibetan sand plover (Charadrius altrifrons) in Qinghai-Tibetan Plateau of China and analyzed the faecal bacterial communities using 16S rRNA sequencing methold. They find a convergence in gut microbial communities between the two species, and also show sex- and age-specific patterns of gut microbiota in the study species. I think it is an important research and the results are reliable and interesting. However, more data and descriptions should be provide and the manuscript need further improvements. In the methods section, the processing method for sequencing data needs to be supplemented with detailed information about software, parameters.In the results section, the analysis should also take into account the factor of sequence abundance for a comprehensive analysis. The authors should discuss influence of diet and habitat on the differences in gut microbiota by using more dietary data and distribution data in this study . More suggestions are as followings:

L56: "the potential to infect human" can be expressed as "the risk of human infection".

L82-84: The phrase "interactions between sex hormones and microbial communities" is inaccurate. It should be the influence of sex hormones on gut microbiota. The cited literature does not prove the impact of microbes on hormones.

L114-116: The hypoxic and cold environmental conditions of the study site were not described earlier in the text. Relevant descriptions should be added before this section.

L123-128: In the hypotheses, the term "differences" refers to specific patterns of divergence or merely the presence of divergence?

L136-138: How to handle the potential bias in differences in sample sizes between the two species?

L160-168: In the sequence alignment processing method, there is a lack of information regarding the software, sequence filtering threshold criteria, and the number of sequences obtained.

L212：Figure 3C should be Figure 2C?

L223-224: Is there any references for determining the criteria for the "core microbiota"?

L262-263: Is there dietary data to support that ‘juvenile prefer to more proportion of vegetations in their diet.’？

L280-283: Besides the same breeding area, could the similar gut microbiota composition between the two species also be attributed to their close genetic relationship?

L296-298: It does not specifically explain the differences in habitat between the Kentish plover and the Tibetan sand plover. Maybe the authors can provide more descriptions here.

L311-313：Please check the citation: ‘a more male biased genus Catellicoccus ’

L305-313: What is the basis for the statement that "the female might be exposed to a more variable bacterial environment"? the authors need clarified.

**Editor Decision: Major Revision**

**Author's Response to Decision Letter for (FEMSMC-2024-008)**

Reviewer 1‘s comments

Reviewer: 1

Comments to the Author

In this paper the authors aimed to understand how host species, sex, and age influence the gut microbiome of two plover species. Whilst they identified few differences amongst species, they identified a sex by age interaction effect. This topic is of interest but not particularly novel- plenty of papers have investigated these questions in bird species and have utilized far greater sample sizes than presented here. I also have several methodological/statistical concerns that would need to be addressed prior to publication.

We appreciate the evaluation.

Major comments

1. It is unclear to me how the results have been analysed. The authors state (line 171) that faecal samples were classified into three groups- Kentish plover (KP) vs Sand plover (SP); female KP vs male KP; adult KP vs juvenile KP. However, it is not clear from the methods, results, Figures or Table legends whether separate analyses were carried out for each of these groups in turn or in some other way.

Thank you for the comments. We add an explanation about the comparison groups in statistical analysis from Line187- 191.

Further to this, the authors have extremely low sample sizes, both for sand plovers (just 1 sample each for male and female juveniles and not much better for adults) and for Kentish plovers (e.g. only 2 juvenile females). I don’t believe they have the power to test for an age*sex interaction at all. A common rule of thumb in statistics is that you should have 10 data points for each predictor term you include in your model; as there are only 33 samples in total (and 24 for KP if analysing on its own) this means at most you could include 2-3 terms in the model before it becomes over-parameterised. Including too many terms may give erroneous results.

In light of this, I would suggest the authors simplify their analyses. Perhaps they could first run an analysis using adult KP and adult SP to see if there are differences across species (using species as a predictor whilst also controlling for sex in the same model). Then use KP samples to test for the effects of age and sex in a second model. These two analyses should be done for alpha and adonis analyses.

We appreciate the pointing out the issue of an insufficient sample size that may cause in the statistical analysis. The result of simpler models using less factor was uploaded in supplementary file3 (sheet 1 for ANOVA test in alpha diversity, sheet 2 for Adonis test in beta diversity). When we comparing the alpha diversity alpha (ANOVA) using the KP sample only, it did not show a significant result (except in Shannon index, the p-value is 0.07, detail information in sheet1 of supplementary file3 ). But given that we only got 2 samples for female juvenile, we could not conclude there is no interaction between the sex and age group. Then we run the Manny-Whitte U test to compare the diversity between female and male in adult KP (sheet1 of supplementary file3 ). In Shannon and Simpson index, the p-value was almost significant (0.07024 and 0.08782) which was acceptable based on the small sample size. This result is consistent with the previous result (Figure 2B). Here, the meaning of the ANOVA test was to show the heterogeneity between adults and juveniles may cover the difference between two sexes.

For the beta diversity, the result of Adonis test using simpler model was uploaded, most significant result came from the model using all the samples comparing the age group (sheet2 of supplementary file3). But when we used only KP samples, there was no significant difference on microbiome community between the adult and juvenile (sheet2 of supplementary file3). Although according the observation in the wild, the color of the facet sample was totally different: adults produced facets in dark brown while the juvenile produced facet in green. Therefore, further sample need to be collected to offer valid evidence showing the diverse microbiome community occurs in different life stage.

2. How did the authors control for differences in sequencing depth across samples? A common practice would be to rarefy reads (i.e. normalize to same read depth) prior to alpha diversity calculations. Otherwise, libraries with greater numbers of reads could generate greater diversity scores. This would be a problem if read depth is confounded with a variable of interest (e.g. if SP samples consistently have lower library sizes than KP, or juveniles have lower read depth than adults). I refer them to this recent publication for an in depth explanation of this: https://journals.asm.org/doi/10.1128/msphere.00355-23 . I would suggest that the authors check whether their results are consistent when they rarefy reads.

Thank you for the suggestion. The amounts of sequencing reads was provided in Supplementary Table 1 where no outliers was detected by boxplot. And rarefaction sampling was conducted when calculating the diversity by sampling 39766 reads each sample (added in the method Line 195). Further, according to the goods coverage index, we could consider the sequencing method was reliable enough to describe the microbiome community (Supplementary Table 3).

Minor comments:

1. Line 41-42: The results presented here are not strongly linked to conservation. I would remove the final part of this sentence alluding to that.

We appreciate the correction. Sentence related to conservation has been deleted (Line 40-42).

2. The second paragraph of the introduction is extremely long. I would suggest splitting into new paragraphs 1) when sex is introduced as a factor (line 71), 2) when development is introduced (line 87), 3) when study species is introduced (line 103)

Thanks for suggestion. The long paragraph has been separated into three short paragraphs.

3. Line 82- mice and cows don’t have cloacas. Do you mean the vaginal microbiome? Or reproductive tract microbiome?

Thanks for pointing this out. We have modified the sentence, please see Line83.

4. Line 95- incomplete maturation of the host immune system or gut morphology in juveniles could also be a factor leading to differences between juvenile and adult microbiota.

We appreciate your insightful comment. We have added this information to our manuscript, please see lines 103-106.

5. Were any controls collected at the time of sampling (e.g. from collectors hands, empty faecal bags etc..). Also, were any negative extraction or PCR blanks included in the sequencing run? These controls are very important in microbiome studies where contaminants could be introduced during sample collection or from laboratory agents leading to elevated diversity and spurious differences across samples.

We agree with you on this. Unfortunately, we did not collect any samples from collector’s hands or faecal bags as a control group. However, we conduct several operations to prevent the contamination and make sure the sample represent the true microbiome community. The process of fecal sampling was referenced in the Line 153. To summarize the sampling process, the birds would be put in a paper bag with plastic rack (preventing the birds contaminates the sample) which were sterilized before with bleach solution. During the process, researcher was wearing sterile gloves and mask and operating a sterile polyester swabs, placed the sample in sterile cryotubes without medium. After the sampling, gloves, mask and paper bag would be deposited, but the plastic rack could be reused after cleaning and sterilization. Several decent studies also apply this method when collecting the faecal samples in wild birds.(Davies, et al. 2022: 41, Grond, et al. 2023: e01128-22, Michel, et al. 2018: 1-14)

During the laboratory work, samples with high quality to build up the library were kept in the study. Several samples with low DNA amount and ineligible DNA concentration would be removed.

Davies CS, Worsley SF, Maher KH et al. Immunogenetic variation shapes the gut microbiome in a natural vertebrate population. Microbiome 2022;10: 41.

Grond K, Louyakis AS, Hird SM. Functional and compositional changes in the fecal microbiome of a shorebird during migratory stopover. mSystems 2023;8: e01128-22.

Michel AJ, Ward LM, Goffredi SK et al. The gut of the finch: uniqueness of the gut microbiome of the Galápagos vampire finch. Microbiome 2018;6: 1-14.

6. Line 188 and 201- OUT should be OTU

Thank, the typo has been corrected.

7. Line 189- “cloacal swabs” should be changed to “faecal samples” as these imply different sample types. I believe you collected the latter

Sorry for the confusion, we indeed used faecal samples, we have made the change, see Line 211.

8. Figure 1- perhaps consider labelling adult/juvenile and male/female samples in the plot for clarity (e.g. by ordering bars and adding facets).

Thanks, we have replaced the figure labels as suggested.

9. Figure 1 c-e How were the taxa selected for these heatmaps/comparisons? Also, lines 206-226 (particularly line 215 “no significant variance between sexes”): heatmaps are not an appropriate method to statistically compare abundances of bacteria between samples and should only be used for visualisation following a statistical differential abundance test e.g. an ANCOM analysis.

Metastats was applied to identify the differential abundant phyla, but no statistical significant result were found possibly due to the limited sample size. Therefore, we decided to visualize the phylum with both biological meaning (according to the published research the microbiome may source from food, environment, or disease, more information in discussion) according to the rank of relative abundance ratio. The statistical result will be uploaded as supplementary file (Supplementary file 2) with the revised manuscript.(Song, et al. 2020: 40)

Song Z, Lin X, Que P et al. The allocation between egg size and clutch size depends on local nest survival rate in a mean of bet-hedging in a shorebird. Avian Research 2020;11: 40.

10. Line 222- was the core microbiome calculated across both species combined, or for KP and SP separately? Differences in sample size would mean the latter is most appropriate. Please clarify in the text.

Thank you for pointing out the inaccurate expression. The definition of core microbiome is the OTUs occur in over 50% of all samples including two species. To clarify, we changed to use the phrase shared core microbiome to infer the definition (Line 192). The reason for choosing this definition is, firstly, we introduced the core microbiome to identify the most persistent microbiome which may be essential for local adaptation and make up the backbone of the gut microbiome community for the birds living in the high-altitude plateau. Secondly, the previous study on arctic shorebirds, also showed consistency in the shared core microbiome among the species living in the same area (referenced in the manuscript Line 113) (Grond, et al. 2019: 2258). Consequently, we assume the shared core microbiome is mainly decided by the local environment which decides the diet and habitat. And our results show the shared core microbiome community differs between the adults and juveniles, indicating the relatively unstable microbiome community in the early stage of life.

Grond K, Santo Domingo JW, Lanctot RB et al. Composition and Drivers of Gut Microbial Communities in Arctic-Breeding Shorebirds. Frontiers in Microbiology 2019;10: 2258.

11. Figures 3 & S2- please add all raw data points to box plots to show the spread of the data- particularly important as you have low sample sizes.

Thanks, we have added the data points to the box plot (Figure3,Supplementary Figure2, Supplementary Figure3).

12. Figure 4- Why did the authors only include taxa with an LDA of >2 (from LEFSE analysis) in this plot? That would give the appearance of greater differentiation than there actually is across samples. Also where are the results of the LEFSE analysis reported (this is linked to my point 9 above- what differential abundance tests were carried out)?

In both LEFSE and Metastats analysis, there is no statistically significant OTUs within each comparison group (result file will be uploaded). And using all 4046 OTUs to run the PCoA analysis, each comparison group did not separately distribute (see Supplementary Figure 4), but according to the Adonis test (Supplementary File3), there is a significantly difference in some groups (i.e. Age). To provide a clearer visualisation, we choose the OTUs mostly contribute to the difference in the PCoA analysis. However, according to the result, this method didn't change the sample distribution a lot but only improved the proportion of variance explained, then finally we decided to adopt this method which gives a better explanation of variance.

13. Line 237- incorrect figure referenced- change FigS1 to FigS2.

Thank you for pointing out the mistake. The correct reference was updated on Line 257.

14. Line 250- incorrect figure referenced- change FigS2 to FigS3?

Thank you for pointing out the mistake. The correct reference was updated on Line 262.

Reviewer 2‘s comments

Comments to the Author

Gut microbiotas affects the reproductive success and survival of birds.In this manuscript, the authors collected samples from two breeding populations of Kentish plover (Charadrius alexandrinus) and Tibetan sand plover (Charadrius altrifrons) in Qinghai-Tibetan Plateau of China and analyzed the faecal bacterial communities using 16S rRNA sequencing methold. They find a convergence in gut microbial communities between the two species, and also show sex- and age-specific patterns of gut microbiota in the study species. I think it is an important research and the results are reliable and interesting.

We appreciate your positive evaluation.

However, more data and descriptions should be provide and the manuscript need further improvements. In the methods section, the processing method for sequencing data needs to be supplemented with detailed information about software, parameters.

More details of sequencing analysis were added from Line 171-183.

In the results section, the analysis should also take into account the factor of sequence abundance for a comprehensive analysis.

In the study, microbiome abundance and diversity were estimated using the OTU matrix under rarefaction in 39766 reads which eliminates the effect of sequencing depth (Line 194).

The authors should discuss influence of diet and habitat on the differences in gut microbiota by using more dietary data and distribution data in this study. More suggestions are as followings:

L56: "the potential to infect human" can be expressed as "the risk of human infection".

Thank you for helping improve the expression. We had modified in Line 56.

L82-84: The phrase "interactions between sex hormones and microbial communities" is inaccurate. It should be the influence of sex hormones on gut microbiota. The cited literature does not prove the impact of microbes on hormones.

Sorry for the ambiguity. We have improved the expression, please see Line 83.

L114-116: The hypoxic and cold environmental conditions of the study site were not described earlier in the text. Relevant descriptions should be added before this section.

Thanks, we have added the relevant information, please see Line 142-145.

L123-128: In the hypotheses, the term "differences" refers to specific patterns of divergence or merely the presence of divergence?

We appreciate the comments. We expand the description in the manuscript, please see Line 129-138. To summarize, in the different comparison groups, we would expect specific divergence pattern when we focus on the composition of the microbiome community and the presence of divergence when looking at the diversity.

L136-138: How to handle the potential bias in differences in sample sizes between the two species?

Thank you for the comments. We do be aware the relatively small sample size of our study. Unfortunately, we are not able to expand the sample size for now. But we adapt several steps to mitigate the bias: (1) our statistical method will take the sample size into account i.e ANOVA, Metastats, etc. to discriminate the difference; (2) we are cautious when interpreting the result, especially the result related to juveniles; (3) we find previous research to support our result and conclusion (Line 306).

L160-168: In the sequence alignment processing method, there is a lack of information regarding the software, sequence filtering threshold criteria, and the number of sequences obtained.

We appreciate the comments. The method of data processing was improved with more details, from Line 160. The details of statistical analysis were also updated from Line 180. The overall statistic of quality of data was described in Supplementary Table 1.

L212：Figure 3C should be Figure 2C?

Thank you for pointing out the mistake. It has been changed on Line 222.

L223-224: Is there any references for determining the criteria for the "core microbiota"?

Yes, please see the reference in Line 193. To make it clearer, we used the phrase "shared core microbiota" to indicate the core microbiome which an essential for the shorebirds living in the Tibet plateau.

L262-263: Is there dietary data to support that ‘juvenile prefer to more proportion of vegetations in their diet.’？

We appreciate the comments. We did not have dietary data in our study. But study about diet in shorebird juveniles was referenced to support our result in Line 356.

L280-283: Besides the same breeding area, could the similar gut microbiota composition between the two species also be attributed to their close genetic relationship?

We appreciate the comments. According to the previous study, we believe that it is more possible that the gut microbiome of avian species was more related to the host physiology condition, diet, and outside environment rather than the phylogenetic relationship(Capunitan, et al. 2020: 829-47). Although these two species are closely related in phylogeny, it's more likely to regard this similarity as the result of holding similar life history and overall habitat near Qinghai Lake.

Capunitan DC, Johnson O, Terrill RS et al. Evolutionary signal in the gut microbiomes of 74 bird species from Equatorial Guinea. Molecular Ecology 2020;29: 829-47.

L296-298: It does not specifically explain the differences in habitat between the Kentish plover and the Tibetan sand plover. Maybe the authors can provide more descriptions here.

Thank you for the comments. We add description and a supplementary figure showing the nest point of Kentish Plover and Tibetan Sand (Supplementary Figure 5), Line 323-326.

L311-313：Please check the citation: ‘a more male biased genus Catellicoccus ’

We appreciate the comments. In this study, the result of Figure 5, the log2 ratio of Catellicoccus abundance between males and females supports our result(Góngora, et al. 2021: 1200).

L305-313: What is the basis for the statement that "the female might be exposed to a more variable bacterial environment"? the authors need clarified.

Thank you for pointing out the unclear expression. More study was cited to explain the reason why females may occur in more diverse environments (Line 335-337).

Reference

Capunitan DC, Johnson O, Terrill RS et al. Evolutionary signal in the gut microbiomes of 74 bird species from Equatorial Guinea. Molecular Ecology 2020;29: 829-47.

Davies CS, Worsley SF, Maher KH et al. Immunogenetic variation shapes the gut microbiome in a natural vertebrate population. Microbiome 2022;10: 41.

Góngora E, Elliott KH, Whyte L. Gut microbiome is affected by inter-sexual and inter-seasonal variation in diet for thick-billed murres (Uria lomvia). Scientific Reports 2021;11: 1200.

Grond K, Louyakis AS, Hird SM. Functional and compositional changes in the fecal microbiome of a shorebird during migratory stopover. mSystems 2023;8: e01128-22.

Grond K, Santo Domingo JW, Lanctot RB et al. Composition and Drivers of Gut Microbial Communities in Arctic-Breeding Shorebirds. Frontiers in Microbiology 2019;10: 2258.

Michel AJ, Ward LM, Goffredi SK et al. The gut of the finch: uniqueness of the gut microbiome of the Galápagos vampire finch. Microbiome 2018;6: 1-14.

Song Z, Lin X, Que P et al. The allocation between egg size and clutch size depends on local nest survival rate in a mean of bet-hedging in a shorebird. Avian Research 2020;11: 40.

**Reviews of manuscript FEMSMC-2024-008.r1**

**Reviewer 1**

**Recommendation: Minor Revision**

The authors have addressed my previous comments satisfactorily. I have a few remaining minor comments that would need to be addressed prior to publication:

Line 104: “very” is an overstatement. There are more studies on mammals but the number of bird microbiome studies is increasing. I would change “very” to “more”

Line 112: define acronyms

Line 124-126: why do expect differences at the phylum level specifically? Need to justify this statement

Line 131: why do you expect lower diversity in juveniles. Hypothesis needs short justification.

Line 188: why was 39866 chosen (is this the minimum read depth)?

Line 251: what do you mean by comparison group- do you mean differences between host species?

Lines 256-261: wrong figure is referenced in this section. Should be “3b” not “2b”

Lines 276-281 should be in the discussion not the results section

Section 284-321: I think the authors need to state somewhere in these paragraphs that there have been very few studies looking at the functionality of specific bacteria in the gut microbiome of birds. Thus, a lot of what they say is speculation. Some speculation is fine but it should be stated that further functional studies would be need to confirm the beneficial/pathogenic role of specific genera.

Line 333: start new paragraph when begin to discuss alpha diversity results

Line 356: the study only looked at diversity and did not assess at stability (do you mean evenness?)- longitudinal samples from the same individual would be needed to assess stability. Delete the word stability. This also needs correcting in the abstract.

**Editor Decision: Minor Revision**

**Author's Response to Decision Letter for (FEMSMC-2024-008.r1)**

Reviewer‘s comments

Comments to the Author

The authors have addressed my previous comments satisfactorily. I have a few remaining minor comments that would need to be addressed prior to publication:

We appreciate the evaluation.

Line 104: “very” is an overstatement. There are more studies on mammals but the number of bird microbiome studies is increasing. I would change “very” to “more”

Thank you for the comments. We have modified the sentence, in Line 103.

Line 112: define acronyms

Thank you for the suggestion. We have clarified the acronyms in Line 109-110.

Line 124-126: why do expect differences at the phylum level specifically? Need to justify this statement

We appreciate the comments. Here, we would expect the microbe community is similar in the general taxonomy level. We have improved the expression in Line 126-127.

Line 131: why do you expect lower diversity in juveniles. Hypothesis needs short justification.

Thank you for the suggestion. We have made the clarification in Line 132-134.

Line 188: why was 39866 chosen (is this the minimum read depth)?

Thanks for raising this question. 39866 is 40% of the sample with minimum reads depth after quality control given that we would like to keep randomness in the sample with minimum reads depth. And according to the rarefaction curve, 39866 is enough to characterize the microbe community.

Line 251: what do you mean by comparison group- do you mean differences between host species?

We appreciate the comments. To make it clear, we have added more justification Line 254-255.

Lines 256-261: wrong figure is referenced in this section. Should be “3b” not “2b”

Thank you for point out the mistake. We had corrected the reference in Line 261.

Lines 276-281 should be in the discussion not the results section

Thank you for the suggestion. We have moved this part into the Discussion (Line 350-359).

Section 284-321: I think the authors need to state somewhere in these paragraphs that there have been very few studies looking at the functionality of specific bacteria in the gut microbiome of birds. Thus, a lot of what they say is speculation. Some speculation is fine but it should be stated that further functional studies would be need to confirm the beneficial/pathogenic role of specific genera.

We appreciate your suggestions. We added the needs of more functional study in the discussion Line 366-367. It reads like “even though we had identified several genera were enriched due to specific physiological characteristics, more functional aspects were needed study to make a solid conclusion”

Line 333: start new paragraph when begin to discuss alpha diversity results

Thanks. Please see Line 338.

Line 356: the study only looked at diversity and did not assess at stability (do you mean evenness?)- longitudinal samples from the same individual would be needed to assess stability. Delete the word stability. This also needs correcting in the abstract.

Thank you for your advice. It true we are overstating. We have modified the expression in Line 39 and Line 363.
